# Tissue-resident macrophages regulate lymphatic vessel growth and patterning in the developing heart

**DOI:** 10.1242/dev.194563

**Published:** 2021-02-03

**Authors:** Thomas J. Cahill, Xin Sun, Christophe Ravaud, Cristina Villa del Campo, Konstantinos Klaourakis, Irina-Elena Lupu, Allegra M. Lord, Cathy Browne, Sten Eirik W. Jacobsen, David R. Greaves, David G. Jackson, Sally A. Cowley, William James, Robin P. Choudhury, Joaquim Miguel Vieira, Paul R. Riley

**Affiliations:** 1 Burdon-Sanderson Cardiac Science Centre, Department of Physiology, Anatomy and Genetics, University of Oxford, Oxford OX1 3PT, UK; 2 British Heart Foundation - Oxbridge Centre of Regenerative Medicine, CRM, University of Oxford, Oxford OX1 3PT, UK; 3 Department of Medicine Huddinge, Center for Hematology and Regenerative Medicine and Department of Cell and Molecular Biology, Karolinska Institutet, Stockholm SE-14186, Sweden; 4 Sir William Dunn School of Pathology, University of Oxford, Oxford OX1 3RE, UK; 5 MRC Human Immunology Unit, Weatherall Institute of Molecular Medicine, John Radcliffe Hospital, University of Oxford, Oxford OX3 9DS, UK; 6 Division of Cardiovascular Medicine, Radcliffe Department of Medicine, University of Oxford, Oxford OX3 9DU, UK

**Keywords:** Macrophages, Hyaluronan, Cell adhesion, Cardiac lymphatics, Coronaries, Vessel growth and patterning

## Abstract

Macrophages are components of the innate immune system with key roles in tissue inflammation and repair. It is now evident that macrophages also support organogenesis, but few studies have characterized their identity, ontogeny and function during heart development. Here, we show that the distribution and prevalence of resident macrophages in the subepicardial compartment of the developing heart coincides with the emergence of new lymphatics, and that macrophages interact closely with the nascent lymphatic capillaries. Consequently, global macrophage deficiency led to extensive vessel disruption, with mutant hearts exhibiting shortened and mis-patterned lymphatics. The origin of cardiac macrophages was linked to the yolk sac and foetal liver. Moreover, the *Cx3cr1*^+^ myeloid lineage was found to play essential functions in the remodelling of the lymphatic endothelium. Mechanistically, macrophage hyaluronan was required for lymphatic sprouting by mediating direct macrophage-lymphatic endothelial cell interactions. Together, these findings reveal insight into the role of macrophages as indispensable mediators of lymphatic growth during the development of the mammalian cardiac vasculature.

## Abstract (precis)

**Highlighted Article:** Tissue-resident macrophages are indispensable mediators of lymphatic vessel formation during heart development, and function to remodel the vascular plexus.

## INTRODUCTION

The cardiac vasculature, composed of the coronary circulation and lymphatic vessel network, begins to develop from around mid-gestation, at approximately embryonic day (E)11.5 in the mouse. Lymphatic endothelial cells (LECs) expressing the canonical lymphatic prospero homeobox 1 transcription factor (PROX1), vascular endothelial growth factor receptor 3 (VEGFR3) and lymphatic vessel endothelial hyaluronan receptor 1 (LYVE1), first arise in the vicinity of the sinus venosus (dorsal side) and outflow tract (ventral side) of the murine heart at ∼E12.5 (Klotz et al., 2015). LECs then assemble into a primitive plexus that expands and remodels prenatally in the sub-epicardial layer along the base-to-apex axis and postnatally towards the myocardium, to form an extensive lymphatic system that drains lymph from the heart to enable optimal cardiac function (Flaht-Zabost et al., 2014; Klotz et al., 2015). Defects in lymphatic drainage are associated with heart disease, where an increase in tissue fluid content by as little as 2.5% can lead to a 30-40% reduction in cardiac output (Dongaonkar et al., 2010; Laine and Allen, 1991). Conversely, cardiac lymphatics respond to myocardial infarction by re-activating a lymphangiogenic gene expression programme; therapeutic stimulation of this process enhances resolution of macrophage-driven inflammation, promoting tissue repair (Klotz et al., 2015; Vieira et al., 2018). Together, these findings emphasize the importance of the cardiac lymphatic system and the need for a better understanding of the cellular and molecular mechanisms underlying its development.

The ontogeny of LECs integrating within the heart and other organ-based lymphatics has been the focus of a paradigm shift in recent times, with non-venous endothelial precursors now accepted as an additional source of the lineage (Eng et al., 2019; Gancz et al., 2019; Klotz et al., 2015; Martinez-Corral et al., 2015; Stanczuk et al., 2015; Stone and Stainier, 2019; Ulvmar and Mäkinen, 2016). The precise identity and origin of these non-venous LEC precursors remains somewhat elusive, although genetic lineage-tracing experiments have implicated the Tie2/PDGFB-negative transient embryonic haemogenic endothelium of the yolk sac and, more recently, second heart field-derived progenitors as contributing to cardiac lymphangiogenesis (Klotz et al., 2015; Lioux et al., 2020; Maruyama et al., 2019).

Macrophages are myeloid immune cells strategically dispersed throughout the tissues of the body. They have a vast functional repertoire and emerging plasticity that converges on normal homeostasis and responses to pathology, through mediating inflammation and repair. Macrophages were initially described in sites of physiological cell death within the bulbus cordis of embryonic chick and rat hearts, using light and electron microscopy (Manasek, 1969; Pexieder, 1975; Sorokin et al., 1994). Subsequently, *in vitro* experiments confirmed macrophages as phagocytic cells and, therefore, it was hypothesized that their primary role was to remove debris arising from cell death (Sorokin et al., 1994). Indeed, macrophages are specialized phagocytes with a classical role in engulfing and digesting dying or dead cells, cellular debris and pathogens. Macrophages are also responsible for cytokine production, and act as a source of pro (lymph-)angiogenic factors, such as VEGFA, VEGFC and VEGFD, which in turn support tumour growth, vascularization and dissemination (Qian and Pollard, 2010). In addition to these classical roles in postnatal settings, macrophages have been implicated more recently in organogenesis during embryonic development and tissue regeneration after injury (Aurora et al., 2014; Theret et al., 2019), as well as in the maintenance of arterial tone through regulation of collagen turnover (Lim et al., 2018). In the embryo, macrophages arise initially from the extra-embryonic transient yolk sac and subsequently from alternative sources within the embryo proper, including foetal liver-resident erythro-myeloid progenitors (EMPs) and hematopoietic stem cells (HSCs) (Ginhoux and Guilliams, 2016). Yolk sac-derived macrophages seed most tissue-resident niches, which are maintained through adulthood by self-renewal (e.g. microglia in the brain) or gradually replenished by foetal liver EMP-derived monocytes, with both populations having distinct roles in tissue injury responses (Hoeffel et al., 2015; Lavine et al., 2014; Stremmel et al., 2018). In the developing heart, the outer mesothelial layer, the epicardium, has been proposed as an important signalling axis to recruit primitive yolk sac-derived macrophages to the subepicardial space (Stevens et al., 2016). The reported timescale for embryonic macrophage recruitment coincides with major morphological changes taking place in the forming heart, i.e. chamber septation, endocardial cushion formation and valve remodelling, myocardial growth and maturation, development of the electrical conduction system, and the emergence and expansion of the coronary and lymphatic vasculature. As such, tissue-resident macrophages have been considered as potential key contributors to some of these processes via their functional roles in engulfing dying cells, releasing soluble cytokines, interacting with or recruiting progenitor cells and their potential to transdifferentiate into alternative cell types. Although a functional requirement has been identified for cardiac macrophages in valvular remodelling, normal conduction and coronary development (Hulsmans et al., 2017; Leid et al., 2016; Shigeta et al., 2019), there have been no reported roles during cardiac lymphatic development to date. Given that macrophages contribute to adult lymphangiogenesis within inflammatory, wound healing and tumour microenvironmental settings (Qian and Pollard, 2010; Ran and Montgomery, 2012), as well as in the developing skin (where they define nascent vessel calibre) (Gordon et al., 2010), we sought to investigate the role of tissue-resident macrophages in the developing heart. Here, we demonstrate for the first time that macrophages are essential for cardiac lymphatic growth and remodelling. Macrophages colonized the embryonic heart prior to the initiation of lymphatic expansion, closely associating and interacting with the adventitial surface and leading edges of lymphatic vessels, where they promoted growth and fusion to ensure an adequate coverage over the subepicardial surface. Global genetic ablation of myeloid cells led to hyperplastic and shortened lymphatic vessels in the heart, which failed to branch properly, and to a mis-patterning of the coronary blood vessels. Both extra- and intra-embryonic hematopoietic sources were found to contribute to the tissue-resident macrophage population, and fate mapping, based on canonical myeloid CSF1R and CX3CR1 markers, identified overlapping lineages that contributed to the remodelling of the nascent lymphatic network. In a co-culture model of human lymphatic endothelial cells with human induced pluripotent stem cell-derived macrophages (hiPSC-macrophages), the latter closely associated with tube-forming lymphatic endothelium, where they induced sprouting, replicating our *in vivo* findings. Mechanistically, a direct interaction between LECs and hiPSC macrophages was found to be dependent on macrophage hyaluronan, a linear glycosaminoglycan composed of a repeating disaccharide unit of D-glucuronic acid and N-acetyl-D-glucosamine, previously implicated in cell motility and adhesion during angiogenesis, and in leukocyte trafficking through the lymph, respectively (Jackson, 2019; Johnson et al., 2017; Lim et al., 2018; Savani et al., 2001). These findings significantly increase our knowledge of (lymphatic) vascular biology and provide further insight into the plasticity and diversity of tissue-resident macrophage function during development. Mechanistic insight into the cellular interactions controlling lymphatic vessel formation is potentially of more widespread interest in terms of understanding how to therapeutically modulate macrophages and lymphatic growth during heart disease.

## RESULTS

### Tissue-resident macrophages are closely associated with developing cardiac lymphatics

**Fig. 1.**
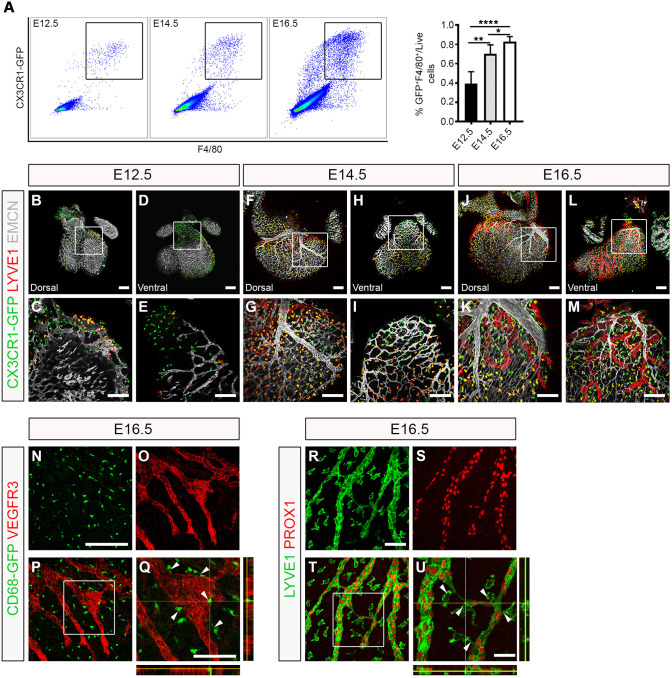
**Tissue-resident macrophages are closely associated with the developing cardiac lymphatics.** (A) Representative histograms and percentage of tissue-resident macrophages over total number of live singlet cells in the developing heart at embryonic days (E) 12.5, E14.5 and E16.5 measured by flow cytometry for GFP and F4/80 in *Cx3cr1^GFP/+^* reporter embryos. GFP^+^F4/80^+^ cells are defined as macrophages. Data are mean±s.e.m.; *n*=6 hearts per group from at least three independent litters. Significant differences (*P*-values) were calculated using one-way ANOVA followed by Tukey's multiple comparison test (*P≤0.05; **P≤0.01; ****P≤0.0001). (B-M) Whole-mount immunostaining for GFP (green), LYVE1 (red) and EMCN (white) to visualize the sub-epicardial tissue-resident macrophages, coronary vessels (capillaries and veins) and lymphatic plexus (respectively) in both the dorsal and ventral aspects of hearts derived from *Cx3cr1^GFP/+^* embryos at E12.5 (B-E), E14.5 (F-I) and E16.5 (J-M). (C,E,G,I,K,M) Magnified views of boxes shown in B,D,F,H,J,L. LYVE1 reactivity is detected in the lymphatic endothelium and tissue-resident macrophages. (N-Q) GFP (green) and VEGFR3 (red) immunostaining of whole-mount hearts derived from *CD68-GFP*-expressing embryos at E16.5. (Q) Orthogonal view of the box shown in P; white arrowheads indicate close association of CD68-GFP^+^ macrophages with VEGFR3-expressing lymphatic vessels. (R-U) LYVE1 (green) and PROX1 (red) immunostaining of whole-mount hearts derived from C57BL6 embryos at E16.5. (U) Orthogonal view of the box shown in T; white arrowheads indicate LYVE1^+^ macrophages interacting with fusing lymphatic tip cells labelled with PROX1 (nuclear) and LYVE1 (membrane). *n*=3-6 hearts per group from at least three independent litters. Scale bars: 100 µm in B-N; 25 µm in Q; 200 µm in R; 50 µm in U.

To investigate the earliest timepoint at which macrophages were first detected in the developing heart, we initially analysed published single-cell RNA sequencing (scRNA-seq) data from whole heart at E9.25 and E10.5 (de Soysa et al., 2019; Hill et al., 2019) (Fig. S1). Although no macrophage cluster was detected at E9.25 (de Soysa et al., 2019), graph-based clustering followed by dimensionality reduction using uniform manifold approximation and projection (UMAP) (Becht et al., 2018) of the E10.5 dataset revealed 18 clusters corresponding to the major cardiac cell types [i.e. cardiomyocytes (CMs), endocardial cells (Endo), mesenchymal cells (Mes), epicardial cells (Epi) and second heart field cardiac progenitor cells (SHF)], which also included resident macrophages (Fig. S1A). The identity of the macrophage cluster was defined by the expression of key myeloid markers, including receptors for the chemokine fractalkine (*Cx3cr1*) and cytokine colony-stimulating factor 1 (*Csf1r*) (Fig. S1B). Expression of the chemotactic ligand *Csf1* was detected exclusively in the Epi cluster, supporting the hypothesis that epicardial signalling underlies cardiac colonization and seeding of myeloid cells in the subepicardial compartment (Fig. S1B) (Stevens et al., 2016). To validate these findings, we made use of the *Cx3cr1-GFP* reporter mouse, which expresses enhanced GFP under the control of the endogenous *Cx3cr1* locus (Jung et al., 2000). We found that GFP^+^ myeloid cells were present in the developing heart at E10.5 ahead of the onset of coronary and lymphatic vessel growth, being located on the sinus venosus and outer surface of the adjacent ventricular wall (Fig. S1C). Next, to assess a possible role in the formation of the cardiac lymphatic system, we measured the numbers and spatial distribution of tissue-resident macrophages in heart specimens at E12.5 and beyond (Fig. 1). Specifically, flow cytometric analysis of *Cx3cr1^GFP/+^* foetal hearts at E12.5, E14.5 and E16.5 combined with staining for the canonical macrophage marker EGF-like module-containing mucin-like hormone receptor-like 1 (EMR1; henceforth, known as F4/80) (Hume and Gordon, 1983) revealed a distinct population of live singlet cells expressing both markers (Fig. 1A). Moreover, the proportion of GFP^+^F4/80^+^ macrophages over the total number of live singlet cells increased gradually from 0.39±0.06 at E12.5 to 0.70±0.05 at E14.5 and 0.83±0.02 at E16.5 (E12.5 versus E14.5, *P*<0.01; E12.5 versus E16.5, *P*<0.001; E14.5 versus E16.5, *P*<0.05; Fig. 1A), suggesting active recruitment and/or proliferation of macrophages throughout the time-course of cardiac development. To further characterize the macrophage population residing in the developing heart of *Cx3cr1^GFP/+^* mice, we performed whole-mount immunofluorescence staining using antibodies against the widely expressed vascular marker endomucin [EMCN; restricted to capillaries and veins from E15.5 onwards (Brachtendorf et al., 2001)] and the lymphatic marker LYVE1, which is also expressed in macrophages (Klotz et al., 2015; Lim et al., 2018) (Fig. 1B-M). At E12.5, GFP^+^ macrophages were found proximal to the sinus venosus (Fig. 1B,C) and in the outflow tract (Fig. 1D,E), prior to the onset of coronary and cardiac lymphatic vessel formation (Chen et al., 2014; Klotz et al., 2015). Indeed, only a primitive EMCN^+^ capillary network and GFP^+^LYVE-1^+^ macrophages, but no LYVE-1^+^ lymphatics, were evident in the subepicardial space at this stage (Fig. 1B-E). At E14.5, GFP^+^LYVE-1^+^ macrophages were more broadly dispersed in both dorsal and ventral surfaces of the developing heart and, in regions covered by vessels, macrophages were found to be in close proximity to, or in direct contact with, the nascent EMCN^+^ coronary and LYVE-1^+^ lymphatic vasculature (compare Fig. 1F,G with Fig. 1H,I). By E16.5, this spatial pattern of distribution was even more evident, with GFP^+^LYVE-1^+^ subepicardial macrophages associating with the contiguous patent coronary and lymphatic networks (dorsal and ventral sides), notably alongside vessel branch junctions, leading ends (i.e. tips) and adventitial surface of vessel walls (Fig. 1J-M). Tissue-resident macrophages have previously been implicated in coronary vessel development and maturation (Leid et al., 2016), but their role in cardiac lymphatic vessel growth and remodelling remains undetermined. To investigate this further, we initially validated the findings in *Cx3cr1^GFP/+^* mice, by investigating the developing cardiac lymphatics in transgenic *hCD68-GFP* mice, which report enhanced GFP in macrophages under the control of the human *CD68* promoter and enhancer sequences (Iqbal et al., 2014), and in wild-type mice combined with VEGFR3, LYVE-1 or PROX1 markers at E16.5 (Fig. 1N-U). Macrophages were found to be colocalized in the subepicardial space with VEGFR3^+^ lymphatic vessels, and were intimately associated with vessel branching and the leading edges of vessel sprouts (Fig. 1Q, white arrowheads and orthogonal views). Moreover, macrophages were detected bridging adjacent PROX1^+^ lymphatic tips potentially to promote vessel fusion (Fig. 1U, white arrowheads), analogous to the previously reported cellular chaperone role for tissue-resident macrophages during blood vessel expansion in the developing hindbrain and postnatal retina (Fantin et al., 2010). Taken together, these studies reveal that tissue-resident macrophages colonize the embryonic heart prior to the formation of the main vascular networks and adopt a spatial distribution in close proximity to, and in contact with, the forming lymphatics.

### Yolk sac-derived myeloid cells associate with lymphatic expansion

**Fig. 2.**
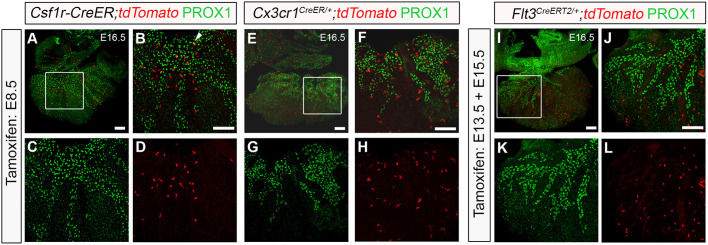
**Yolk sac-derived *Csf1r*^+^ and *Cx3cr1^+^* lineages are associated with cardiac lymphatic growth and expansion.** (A-D) Genetic lineage-tracing based on the activity of the *Csf1r-CreER;tdTomato* transgene induced by tamoxifen administration at embryonic day (E) 8.5. Whole hearts were analysed for Tomato (red) and PROX1 (green) expression at E16.5. (B-D) Magnified views of the box shown in A; white arrowhead indicates colocalization of native Tomato and PROX1 immunoreactivity. (E-H) Genetic lineage-tracing based on the activity of the *Cx3cr1^CreER/+^;tdTomato* transgene induced by tamoxifen administration at E8.5. Whole hearts were analyzed for Tomato (red) and PROX1 (green) expression at E16.5. (F-H) Magnified views of the box shown in E. (I-L) Genetic lineage-tracing based on the activity of the *Flt3^CreERT2/+^;tdTomato* transgene induced by repeated tamoxifen administration at E13.5 and E15.5. Whole hearts were analyzed for Tomato (red) and PROX1 (green) expression at E16.5. (J-L) Magnified views of the box shown in I. *n*=3-6 hearts per group from at least three independent litters. Scale bars: 100 µm.

To define the origins of macrophages colonizing the developing heart during lymphatic vessel development, we employed genetic lineage tracing using *Csf1r*-, *Cx3cr1*- and *Flt3*-based mouse models, which in combination capture the main hematopoietic sources from early to mid-gestation, i.e. the yolk sac (*Csf1r* and *Cx3Cr1*) and foetal liver (*Flt3*) (Benz et al., 2008; Ginhoux and Guilliams, 2016; Gomez Perdiguero et al., 2015; Kasaai et al., 2017; Stremmel et al., 2018) (Fig. 2). We initially employed the *Csf1r-Mer-iCre-Mer* transgenic model (henceforth referred to as, *Csf1r-CreER* mice) (Qian et al., 2011) crossed with *R26R-tdTomato* reporter mice in which CRE recombinase activity downstream of the *Csf1r* promoter sequence was induced by tamoxifen pulsing at E8.5 (Fig. 2A-D). This phased induction ensured mapping of the spatiotemporal pattern of *Csf1r* activity in the transient yolk sac-derived EMP compartment (Gomez Perdiguero et al., 2015; Hoeffel et al., 2015). At E16.5, *Csf1r-CreER^+^* hearts pulsed at E8.5 revealed tdTomato^+^ cells in close proximity to, or in direct contact with, PROX1-expressing LECs, with some of these cells exhibiting dual expression of tdTomato and PROX1 (Fig. 2B, white arrowhead). Of note, this modest, but reproducible contribution of the *Csf1r*^+^ lineage to lymphatic endothelium (see also Klotz et al., 2015), supports the hypothesis that a subset of CSF1R^+^ EMPs colonizing the developing heart may give rise to LECs that integrate the nascent vasculature, contributing to the reported endothelial heterogeneity (Klotz et al., 2015; Lioux et al., 2020; Maruyama et al., 2019). Recently, a yolk sac-derived CSF1R^+^ EMP population was reported to contribute extensively to the endothelium of the nascent vasculature of the developing hindbrain (Plein et al., 2018). However, unlike in the brain, yolk sac-derived CSF1R+ EMPs colonizing the heart appear to give rise mostly to tissue-resident macrophages seeding the subepicardial compartment prior to the onset of lymphatic expansion, i.e. E12.5 (Fig. S2A-D).

To more specifically characterize the ontogeny of cardiac macrophages, given the broader lineage labelling achieved with the *Csf1r-CreER* model (i.e. EMPs, EMP-derived macrophages and EMP-derived LECs), we analysed the hearts of *Cx3cr1^CreER/+^;tdTomato^+^* embryos, pulsed with tamoxifen at E8.5 (Fig. 2E-H). The *Cx3cr1^CreER^* mouse drives expression of the fusion protein CRE-ERT2 (Cre recombinase-oestrogen receptor) under the control of the endogenous myeloid-specific *Cx3cr1* locus (Parkhurst et al., 2013) and hence, tamoxifen pulse-chase from E8.5 was anticipated to capture the involvement of yolk sac-derived pre-macrophages (Ginhoux and Guilliams, 2016; Stremmel et al., 2018). Using this approach, we observed widespread contribution from extra-embryonic hematopoietic compartments (i.e. yolk sac) to the macrophage population residing in the developing heart and closely associated with the nascent PROX1^+^ lymphatic network. Of note, tamoxifen pulsing at E8.5 led to labelling (tdTomato^+^) of 60.5%±4.29% of total LYVE1+ resident macrophages in the developing heart (Fig. S2E-H; *n*=5 hearts). Moreover, we did not observe any direct contribution of the *Cx3cr1*^+^ lineage to LECs within the forming lymphatic plexus, confirming its myeloid cell specificity (compare Fig. 2E-H with Fig. 2A-D).

To investigate a potential contribution from intra-embryonic definitive HSCs to the tissue-resident macrophage population in the developing heart, we used the *Flt3^CreERT2^* mouse model (Benz et al., 2008). Upregulation of FMS-like tyrosine kinase 3 (FLT3; also known as foetal liver kinase 2 or FLK2) is accompanied by loss of self-renewal of definitive HSCs and *Cre* recombinase-driven by the *Flt3* locus has been demonstrated to label all progeny arising from HSCs, including myeloid cells (Boyer et al., 2011). At E16.5, *Flt3^CreERT2/+^* hearts pulsed at E13.5 and E15.5 to maximize recombination efficiency driven by the onset of *Flt3* expression resulted in tdTomato^+^ cells residing in close proximity to, or in direct contact with, PROX1^+^ lymphatic vessels on the subepicardial surface (Fig. 2I-L), indicating cell contribution from the definitive HSC compartment in the foetal liver to the heart at later stages of foetal development when lymphatic expansion is already well-established. Again, as for the *Cx3cr1^+^* lineage, no direct contribution to cardiac lymphatic endothelium was observed. However, closer examination of the *Flt3*^+^ derivatives resident in the heart at E16.5 by immunostaining revealed that these cells lacked the expression of LYVE-1, raising questions about their identity (Fig. S2M-P).

### Macrophages are required for growth and branching of the cardiac lymphatic network

**Fig. 3.**
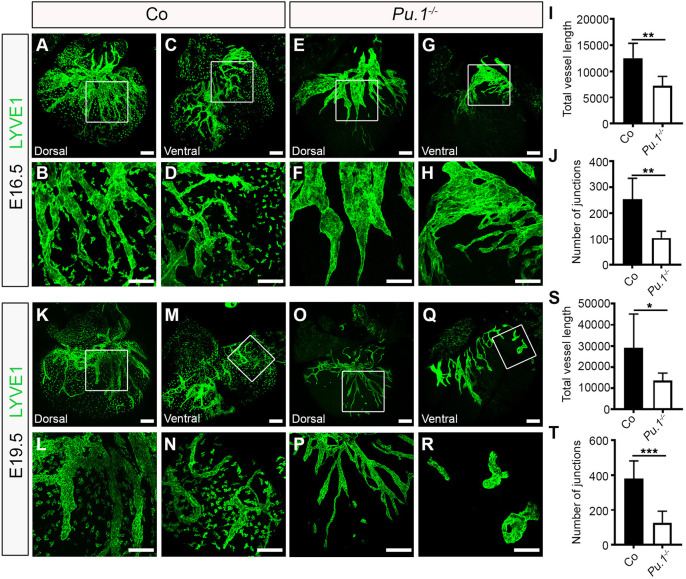
**Macrophages are essential for growth and branching of the cardiac lymphatic network.** (A-H) Whole-mount immunostaining for LYVE1 (green) to visualize the sub-epicardial lymphatic plexus in both the dorsal and ventral aspects of hearts derived from littermate control (co; A-D) or *Pu.1^−/−^* (E-H) embryos at E16.5. (B,D,F,H) Magnified views of boxes shown in A,C,E,G. LYVE1 reactivity is absent in tissue-resident macrophages in *Pu.1^−/−^* hearts compared with control littermates, confirming absence of macrophages. (I,J) Quantification of total vessel length (µm; I) and number of lymphatic vessel junctions (J) in control versus *Pu.1^−/−^* hearts at E16.5. Data are mean±s.e.m.; *n*=6 hearts per group from three independent litters. Significant differences (*P*-values) were calculated using an unpaired, two-tailed Student's *t*-test (**P≤0.01). (K-R) Whole-mount immunostaining for LYVE1 (green) to visualize the sub-epicardial lymphatic plexus in both the dorsal and ventral aspects of hearts derived from littermate control (co; K-N) or *Pu.1^−/−^* (O-R) embryos at E19.5. (L,N,P,R) Magnified views of boxes shown in K,M,O,Q. (S,T) Quantification of total vessel length (µm; S) and number of lymphatic vessel junctions (T) in control versus *Pu.1^−/−^* hearts at E19.5. Data are mean±s.e.m.; control, *n*=5 hearts; *Pu.1^−/−^*, *n*=6 hearts from four independent litters. Significant differences (*P*-values) were calculated using an unpaired, two-tailed Student's *t*-test (*P≤0.05; ***P≤0.001). Scale bars: 100 µm.

**Fig. 4.**
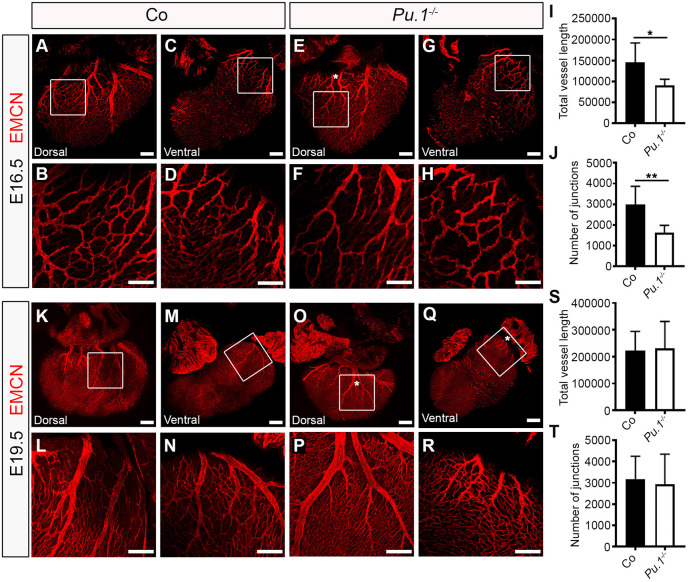
**Macrophages regulate coronary growth and patterning.** (A-H) Whole-mount immunostaining for EMCN (red) to visualize the sub-epicardial coronary vessels (capillaries and veins) in both the dorsal and ventral aspects of hearts derived from littermate control (co; A-D) or *Pu.1^−/−^* (E-H) embryos at E16.5. (B,D,F,H) Magnified views of boxes shown in A,C,E,G. White asterisk indicates patterning defects, i.e. an extra mid-branch, of the coronary veins on the dorsal aspect of *Pu.1^−/−^* hearts (compared E with A). (I,J) Quantification of total vessel length (µm; I) and number of vessel junctions (J) in control versus *Pu.1^−/−^* hearts at E16.5. Data are mean±s.e.m.; *n*=6 hearts per group from three independent litters. Significant differences (*P*-values) were calculated using an unpaired, two-tailed Student's *t*-test (*P≤0.05; **P≤0.01). (K-R) Whole-mount immunostaining for EMCN (red) to visualize the sub-epicardial coronary vessels (capillaries and veins) in both the dorsal and ventral aspects of hearts derived from littermate control (co; K-N) or *Pu.1^−/−^* (O-R) embryos at E19.5. (L,N,P,R) Magnified views of boxes shown in K,M,O,Q. White asterisks indicate patterning defects, i.e. extra branches, of the coronary veins on both the dorsal and ventral aspects of *Pu.1^−/−^* hearts (compare O,Q with K,M). (S,T) Quantification of total vessel length (µm; S) and number of vessel junctions (T) in control versus *Pu.1^−/−^* hearts at E19.5. Data are mean±s.e.m.; control, *n*=5 hearts; *Pu.1^−/−^*, *n*=6 hearts from four independent litters. No significant differences were determined using an unpaired, two-tailed Student's *t*-test. Scale bars: 100 µm in A-D,F-H,K-R; 1 mm in E.

Our data suggest that yolk sac-derived myeloid lineages, defined by the activity of *Csf1r* and *Cx3cr1* gene expression*,* migrate to the heart and give rise to macrophages that seed the outer surface of the ventricular wall and interact closely with the neighbouring lymphatic vessel network that develops from E12.5 onwards. To determine the functional requirements for these tissue-resident macrophages, we first investigated cardiac lymphatic development in a mouse model deficient for the essential ETS domain-containing transcription factor PU.1 (Fig. 3). *Pu.1* (also known as *Spi1*) knockout mice die around birth due to a severely underdeveloped immune system, including a lack of myeloid cells (McKercher et al., 1996; Scott et al., 1994). The number of myeloid cells and macrophages in the developing hearts of *Pu.1*-null mice was determined by flow cytometry for the expression of the pan-leukocyte CD45, myeloid CD11b and macrophage F4/80 markers, and found to be significantly reduced compared with control littermate embryos (CD45^+^CD11b^+^ myeloid cells: 1.35±0.25% versus 0.02±0.008% of all live cells, *P*<0.01; CD45^+^CD11b^+^F4/80^+^ macrophages: 85.4±2.77% versus 0.0±0.0% of all live cells, *P*<0.0001; Fig. S3A-D). As a consequence, cardiac lymphatic growth and patterning was observed to be severely disrupted in *Pu.1*^−/−^ compared with control littermates at E16.5, with mutant hearts exhibiting hyperplastic and shortened LYVE-1^+^ vessels (vessel length: 12,536±1155 µm versus 7231±734.8 µm; *P<*0.01), as well as a significant reduction in the number of junctions (i.e. branchpoints; control 254.2±32.77 versus *Pu.1^−/−^* 104.0±10.52; *P<*0.01) and overall plexus complexity; indicative of a sprouting/branching defect (Fig. 3A-J). A similar phenotype was observed at E19.5, before the onset of embryo demise at perinatal stages (Fig. 3K-T). Embryo size and heart morphology in *Pu.1*-deficient specimens appeared grossly normal, with no evident defects in heart growth, cardiac septation or compaction of the myocardial layer (Fig. S3E,F), hence ruling out the possibility of a secondary cardiac phenotype contributing to the observed lymphatic defects. However, coronary blood vessel growth and patterning were also affected, with null hearts displaying an overall reduction in subepicardial capillary vessel length and junction number (Fig. 4A-J), as well as exhibiting extra branches of main EMCN^+^ coronary veins (Fig. 4E, white asterisk) compared with control littermates at E16.5. The mis-patterning of EMCN^+^ coronary veins was still evident in *Pu.1*-deficient hearts at E19.5 (Fig. 4K-R, white asterisks in O and Q), whereas the coronary capillary vessel length and junction density were restored to levels equivalent to those in control littermate hearts (Fig. 4S,T).

In a previous study, macrophages in the skin were shown to define dermal vessel calibre by regulating lymphatic endothelial cell proliferation (Gordon et al., 2010). However, we did not observe any gross changes in LEC proliferation in *Pu.1*-null hearts compared with control littermates at E16.5 (Fig. S3G-N), suggesting inherent differences across organ-specific lymphatic beds. The latter is supported by the emerging recognition of heterogeneity across the lymphatic vascular system and the existence of distinct origins for subpopulations of LECs (Ulvmar and Mäkinen, 2016).

**Fig. 5.**
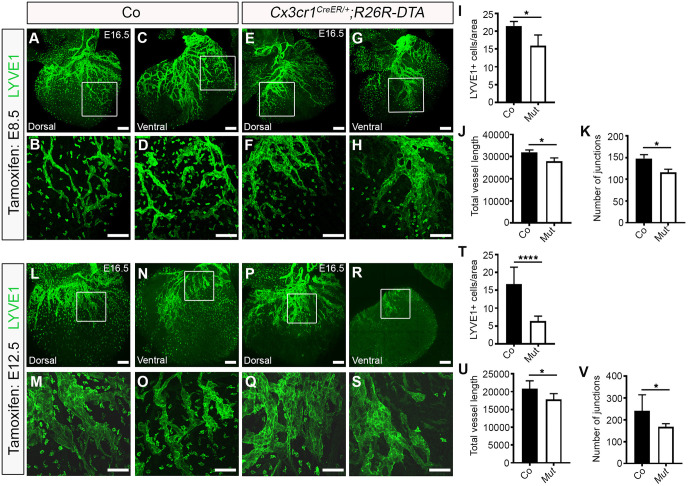
**Ablation of yolk sac-derived macrophages impairs cardiac lymphatic growth and branching.** (A-H) Whole-mount immunostaining for LYVE1 (green) to visualize the sub-epicardial lymphatic plexus in both the dorsal and ventral aspects of E16.5 hearts-derived from littermate control (co; A-D) or *Cx3cr1^CreER/+^;R26R-DTA* (Mut; E-H) embryos that were tamoxifen induced at E8.5. (B,D,F,H) Magnified views of boxes shown in A,C,E,G. (I-K) Quantification of LYVE1^+^ cells per ventricular surface area (I), total vessel length (µm; J) and number of lymphatic vessel junctions (K) in control versus mutant hearts at E16.5. Data are mean±s.e.m.; *n*=3 hearts per group from two independent litters. Significant differences (*P*-values) were calculated using an unpaired, two-tailed Student's *t*-test (*P≤0.05). (L-S) Whole-mount immunostaining for LYVE1 (green) to visualize the sub-epicardial lymphatic plexus in both the dorsal and ventral aspects of E16.5 hearts derived from littermate control (co; L-O) or *Cx3cr1^CreER/+^;R26R-DTA* (Mut; P-S) embryos that were tamoxifen induced at E12.5. (M,O,Q,S) Magnified views of boxes shown in L,N,P,R. (T-V) Quantification of LYVE1^+^ cells per ventricular surface area (T), total vessel length (µm; U) and number of lymphatic vessel junctions (V) in control versus Mut hearts at E16.5. Data are mean±s.e.m.; control, *n*=8 hearts; Mut, *n*=7 hearts from three independent litters. Significant differences (*P*-values) were calculated using an unpaired, two-tailed Student's *t*-test (*P≤0.05; ****P≤0.0001). Scale bars: 100 µm.

**Fig. 6.**
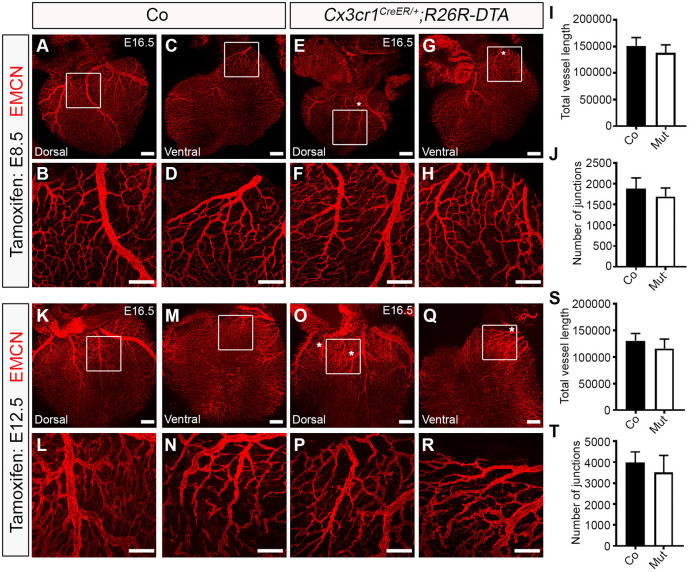
**Ablation of yolk sac-derived macrophages disrupts coronary vein patterning but does not impact on vessel growth or branching.** (A-H) Whole-mount immunostaining for EMCN (red) to visualize the sub-epicardial coronary vessels (capillaries and veins) in both the dorsal and ventral aspects of E16.5 hearts derived from littermate control (co; A-D) or *Cx3cr1^CreER/+^;R26R-DTA* (Mut; E-H) embryos that were tamoxifen induced at E8.5. (B,D,F,H) Magnified views of boxes shown in A,C,E,G. White asterisks indicate patterning defects, i.e. an extra branch, of the coronary veins on the dorsal and ventral aspects of *Cx3cr1^CreER/+^;R26R-DTA* hearts (compared E,G with A,C), akin to *Pu.1*-null hearts (compared with Fig. 4). (I,J) Quantification of total vessel length (µm; I) and number of vessel junctions (J) in control versus mutant hearts at E16.5. Data are mean±s.e.m.; *n*=3 hearts per group from two independent litters. No significant differences were determined using an unpaired, two-tailed Student's *t*-test. (K-R) Whole-mount immunostaining for EMCN (red) to visualize the sub-epicardial coronary vessels (capillaries and veins) in both the dorsal and ventral aspects of E16.5 hearts derived from littermate control (co; K-N) or *Cx3cr1^CreER/+^;R26R-DTA* (Mut; O-R) embryos that were tamoxifen induced at E12.5. (L,N,P,R) Magnified views of boxes shown in K,M,O,Q. White asterisks indicate patterning defects, i.e. an extra branch, of the coronary veins on the dorsal and ventral aspects of *Cx3cr1^CreER/+^;R26R-DTA* hearts (compare O,Q with K,M), akin to *Pu.1*-null hearts (compared with Fig. 4). (S,T) Quantification of total vessel length (µm; S) and number of vessel junctions (T) in control versus mutant hearts at E16.5. Data are mean±s.e.m.; control, *n*=8 hearts; mutant, *n*=7 hearts from three independent litters. No significant differences were determined using an unpaired, two-tailed Student's *t*-test. Scale bars: 100 µm.

To specifically confirm a functional role for yolk sac-derived macrophages in cardiac lymphatic development and to exclude confounding factors arising from the indiscriminate targeting of immune cell development and potentially contributing to the severity of the phenotype in a *Pu.1^−/−^* background, we initially analysed cardiac lymphatic development in mice expressing cytotoxic diphtheria toxin A (DTA) under the control of *Cx3cr1^CreER^* pulsed with tamoxifen at E8.5 (henceforth, *Cx3cr1^CreER/+^;R26R-DTA*). Of note, the use of the *Csf1r-CreER* line was excluded here, because this transgene does not discriminate between bona fide EMP-derived tissue-resident macrophages and EMP-derived LECs (Fig. 2; also see Klotz et al., 2015). At E16.5, hearts isolated from *Cx3cr1^CreER/+^;R26R-DTA* specimens were found to have significantly less tissue-resident macrophages in the subepicardial compartment (control 21.51±0.720 versus mutant 15.96±1.723 LYVE1^+^ cells/ventricular surface area; *P<*0.05) and displayed mild lymphatic vessel hyperplasia, with reduced vessel length growth and junction number, compared with control littermate samples (vessel length: 32,000±577.4 µm versus 28,000±1000 µm; *P*<0.05; number of junctions: 148.0±5.000 versus 116.0±5.000; *P*<0.05; Fig. 5A-K). Next, to enable normal cardiac seeding by yolk sac macrophages and assess the impact of ablating these cells at the onset of lymphatic expansion, we analysed vessel growth and branching in mice pulsed with tamoxifen at E12.5, rather than E8.5. The lineage tracing efficiency in *Cx3cr1^CreER/+^* hearts using this tamoxifen regimen was 92.5%±0.04% (tdTomato^+^LYVE1^+^/total LYVE1^+^ resident macrophages; *n*=5 hearts), with no direct contribution of the *Cx3cr1*^+^ lineage to LECs within the forming lymphatic plexus being observed (Fig. S2I-L). Moreover, in this model, tamoxifen administration at E12.5 is anticipated to target mostly yolk sac-derived macrophages, as CX3CR1 is not expressed in foetal liver-derived monocytes or their precursors (Yona et al., 2013). At E16.5, hearts isolated from *Cx3cr1^CreER/+^;R26R-DTA* embryos pulsed with tamoxifen at E12.5 exhibited considerably less tissue-resident macrophages in the subepicardial layer compared with control littermate samples (control 16.72±1.945 versus mutant 6.349±0.469 LYVE1^+^ cells/ventricular surface area; *P<*0.0001; Fig. 5L-T), and consequently lymphatic vessel growth was found to be disrupted, with vessel length and number of junctions (i.e. vessel branchpoints) significantly reduced (vessel length: 20,866±1088 µm versus 17,800±734.8 µm; *P*<0.05; number of junctions: 240.9±26.02 versus 168.0±5.542; *P*<0.05; Fig. 5U,V). This phenotype, although significant, was milder than that observed in *Pu.1*-null hearts and may be explained by the efficiency of the transient ablation (tamoxifen-induced) of macrophages in *Cx3cr1^CreER/+^;R26R-DTA* embryos, as well as the concomitant replacement of yolk sac-derived macrophages by foetal liver-resident, EMP-derived monocytes and definitive HSC-derived blood-borne monocyte progenitors. This macrophage replacement occurs throughout the time-course of lymphatic vessel development and during this experiment following ablation of the *Cx3cr1^+^* lineage. The compensation by further seeding of tissue-resident macrophages is not a factor in *Pu.1* mutants, which permanently lack the entire myeloid lineage (compare Fig. 3F,H,P,R, devoid of any resident macrophages in *Pu.1*-null hearts, with Fig. 5F,H,Q,S, where there is evidence of residual or replenished LYVE-1+ macrophages in *Cx3cr1^CreER/+^;R26R-DTA* mutant hearts). Interestingly, genetic deletion of the *Cx3cr1^+^* lineage resulting from tamoxifen pulsing at either E8.5 or E12.5 also recapitulated the mis-patterning of the main coronary veins observed in *Pu.1^−/−^* hearts (Fig. 6A-T; white asterisks in E,G,O,Q). Collectively, these data suggest a hitherto unidentified function for tissue-resident macrophages in regulating the growth, patterning and expansion of the contiguous cardiac lymphatic system.

### Macrophage hyaluronan promotes lymphatic cell network formation and sprouting

**Fig. 7.**
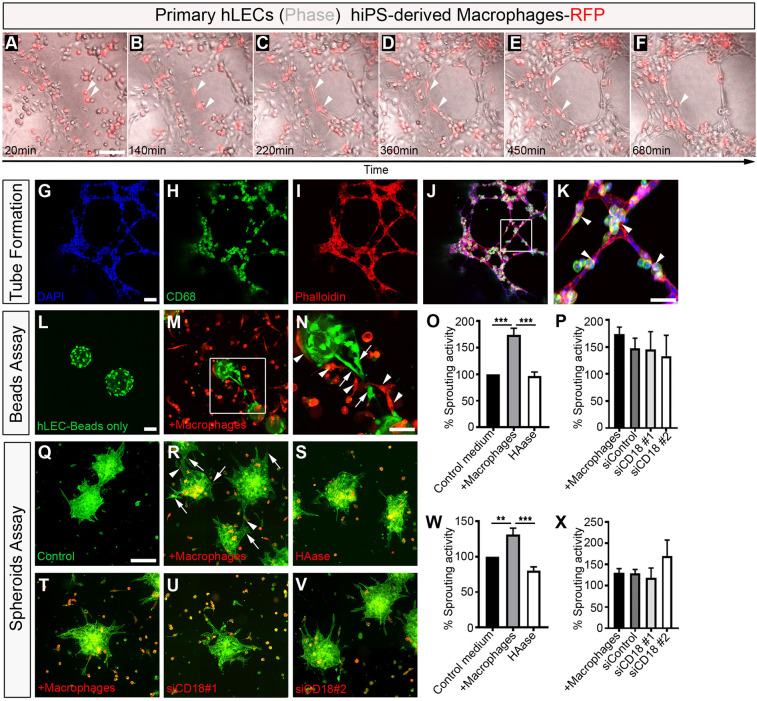
**Macrophages promote lymphatic cell plexus formation and sprouting.** (A-F) Representative frames of time-lapse experiments using primary human lymphatic endothelial cells (hLECs; phase-contrast) co-cultured with human iPS-derived macrophages labelled with RFP (red), showing close association between macrophages and LECs, and a change in macrophage cell morphology (white arrowheads) contacting the expanding lymphatic plexus to facilitate LEC tube formation. (G-K) DAPI (blue), CD68 (green) and phalloidin (red) staining of primary hLEC and hiPSC-macrophage co-cultures. (K) Magnified view of box shown in J. Arrowheads indicate macrophages. (L-N) Representative staining of lymphatic capillary sprouting from beads coated with primary hLECs only (green; control medium; L) or co-cultured with hiPSC-derived macrophages-RFP (red; +macrophages; M). (N) Magnified view of the box shown in M. Arrows and arrowheads indicate lymphatic sprouts and macrophages, respectively. (O,P) Quantification of the percentage sprouting activity defined as (number capillary sprouts/number of beads)×100. Data are normalized against the control group. Data are mean±s.e.m.; control, *n*=6; +macrophages, *n*=6; HAase 30 U/ml, *n*=3; siControl, *n*=3; siCD18 #1, *n*=3; siCD18 #2, *n*=3 independent experiments, with three replicates/experiment. Significant differences (*P*-values) were calculated using one-way ANOVA followed by Tukey's multiple comparison test (***P≤0.001). (Q-V) Representative staining of lymphatic capillary sprouting from hLECs aggregates/spheroids only (green; control medium; Q) or co-cultured with hiPSC-derived macrophages-RFP (red; +macrophages; R,T); and of hiPSC-derived macrophages-RFP pre-treated with HAase 30 U/ml (S) or co-cultured with hiPSC-derived macrophages-RFP pre-treated with siRNA oligonucleotides against *ITGB2*/*CD18* (siCD18 #1, U; siCD18 #2, V). (W,X) Quantification of the percentage of sprouting activity defined as (number capillary sprouts/number of beads)×100. Data were normalized against the control group. Data are mean±s.e.m.; control, *n*=5; +macrophages, *n*=4; HAase 30 U/ml, *n*=4; siControl, *n*=3; siCD18 #1, *n*=3; siCD18 #2, *n*=3 independent experiments, with three replicates/experiment. Significant differences (*P*-values) were calculated using one-way ANOVA followed by Tukey's multiple comparison test (**P≤0.01; ***P≤0.001). Scale bars: 100 µm in A-M,R-V; 50 µm in N; 250 µm in Q.

The close proximity of tissue-resident macrophages to the developing cardiac lymphatics and evident association with the forming lymphatic endothelium suggests their role in regulating growth and patterning may be mediated by cell-cell contact at the leading edges of fusing branches or the adventitial surface of vessel walls (Figs 1–6). To further explore this possibility, we modelled LEC-macrophage interactions during lymphatic capillary tube formation and sprouting in a human *in vitro* setting, by co-culturing human primary lymphatic endothelial cells and human induced pluripotent stem cell (hiPSC)-derived macrophages labelled with a red fluorescent protein (RFP) reporter (Fig. 7; Movie 1). The hiPSC-derived RFP^+^ macrophages used here have been previously characterized and shown to exhibit foetal-like properties, mimicking the expression profile and function/activity of tissue-resident macrophages (Buchrieser et al., 2017; Haenseler et al., 2017; van Wilgenburg et al., 2013). In tube formation assays, hiPSC-RFP^+^ macrophages were found in close proximity to, and associated with LECs, changing cell shape to apparently guide LEC extension and fusion to a neighbouring LEC, leading to vessel-like structures (Fig. 7A-F; white arrowheads), analogous to the behaviour we observed for embryonic macrophages in the developing mouse heart (Fig. 1). Time-lapse live imaging revealed this to be a highly dynamic process, with individual RFP-labelled cells uncoupling from an individual LEC once a tube was formed and scanning the neighbouring micro-environment for further LECs undergoing an equivalent transformation (Movie 1). Likewise, coincident with most of the tube-like structures becoming organized into a plexus, hiPSC-macrophages expressing CD68 were found to be directly associated with the forming tubes and branching nodes (Fig. 7G-K), phenocopying the behaviour observed *in vivo* for tissue-resident embryonic macrophages interacting with cardiac lymphatics (e.g. Fig. 1Q). To model lymphatic sprouting, as an essential first step in lymphatic growth and patterning, we employed two independent three-dimensional (3D) assays based on co-culturing primary human LEC-coated microbeads (hereafter referred to as the beads assay) or aggregates of human LECs (the spheroids assay) with iPSC-derived macrophages (Fig. 7L-X) (Schulz et al., 2012). In the former assay, co-culturing LEC-coated microbeads with macrophages led to a significant increase in the number of lymphatic-like sprouts per micro-bead, compared with control culture conditions with macrophage:LEC media alone, with RFP-expressing macrophages adjoining and in direct contact with the sprout-leading LEC (control conditions, 100% versus +macrophages, 173.7±12.90% sprouting activity; *P*<0.001; Fig. 7L-P). A similar increase in lymphatic sprouting was observed in the spheroids assay (control conditions, 100% versus +macrophages, 130.9±9.387% sprouting activity; *P*<0.01; Fig. 7Q-X). To gain insight into the molecular mechanism(s) underpinning stimulation of LEC sprouting by macrophages, we considered paracrine secretion of VEGFC, a potent lymphangiogenic factor known to be expressed by cardiac macrophages in a myocardial infarction setting (Vieira et al., 2018), and cell-adhesion molecules previously implicated in leukocyte-endothelium interactions during immune response, specifically integrin subunit β2 (ITGB2; also known as CD18)- and hyaluronan (HA)-dependent pathways (Gahmberg, 1997; Jackson, 2019; Johnson et al., 2017). Although *VEGFC* expression was undetectable, iPS-derived macrophages expressed both *ITGB2* and *ITGAM* (also known as CD11b), the heterodimeric components of the essential macrophage antigen 1 (MAC1) receptor that binds to endothelial intracellular adhesion molecule 1 (ICAM1) during leukocyte arrest, rolling and transmigration across the blood vessel wall (Gahmberg, 1997). Notably, they also exhibited a dense coat of HA (previously termed the HA glycocalyx; Johnson et al., 2017; Lawrance et al., 2016) and expressed the HA-binding receptors *LYVE1*, *CD44* and *HMMR* (hyaluronan-mediated motility receptor; also known as CD168) (Fig. S4A-D). To discriminate between potential HA- and CD18-mediated adhesion to endothelium, we pre-treated iPSC-derived macrophages with hyaluronidase (HAase), to deplete surface glycocalyx-associated HA (Johnson et al., 2017; Lawrance et al., 2016), or with siRNA oligonucleotides targeting *ITGB2/CD18*, to specifically knockdown expression of this β-integrin subunit without affecting the related *ITGAM* expression (Fig. S4B-F). Neither treatment had a negative effect on macrophage viability. We found that loss of macrophage *ITGB2/CD18* had no effect on sprouting activity, but macrophage pre-treatment with HAase significantly impaired LEC sprouting in both bead and spheroid sprouting assays (beads assay: +macrophages, 173.7±12.90 versus HAase, 96.90±7.205% sprouting activity; *P<*0.001; spheroids assay: +macrophages, 130.9±9.387 versus HAase, 80.11±5.588% sprouting activity; *P<*0.001; Fig. 7O-X), indicating a requirement for macrophage hyaluronan in human lymphatic endothelial growth. Importantly, yolk sac-derived macrophages residing in the HA-rich layer of the epicardium/subepicardium compartment of the developing mouse heart appear to lack VEGFC expression and displayed membrane-bound HA (Fig. S5). Taken together with our *Pu.1^−/−^* and *Cx3cr1^CreER/+^;R26R-DTA* mutant analyses (Figs 3 and 5), these findings suggest a crucial requirement for an evolutionarily-conserved (mouse to human) HA-dependent mechanism underpinning direct cell-cell contact/adhesion between macrophages and LECs during vessel extension and remodelling.

## DISCUSSION

Here, we reveal a novel function for macrophages residing in the developing heart as lymphatic vessel ‘remodellers’ (Figs 1-7). Our findings indicate that primitive macrophages migrate from the yolk sac to the developing heart between E9.25 to E10.5 to initially colonize the outer surface of the ventricular wall, where they reside in the epicardium/subepicardium compartment prior to cardiac lymphatic vessel formation. This compartment has previously been suggested as an essential signalling axis; whereby epicardial disruption, downstream of Wilms' tumour 1 (*Wt1*) gene deficiency, impaired cardiac recruitment of yolk sac macrophages (Stevens et al., 2016). However, the identity of the epicardial signal(s) required for this process has remained elusive. scRNA-seq analysis of E10.5 hearts (Fig. S1) revealed that expression of the cytokine *Csf1* was exclusively expressed in epicardial cells. CSF1 regulates the differentiation of most macrophage populations, including primitive yolk sac derived, and is a potent macrophage chemoattractant (Ginhoux et al., 2010). Therefore, CSF1 is a likely mediator of macrophage recruitment into the subepicardial region of the developing heart. Future studies investigating the control of *Csf1* activity by epicardial factors such as WT1 may provide insight into the molecular basis leading to cardiac seeding by yolk sac-derived macrophages.

The epicardium plays an essential role during heart development by secreting paracrine signals stimulating cardiomyocyte proliferation and maturation, as well as supporting cardiac vasculature expansion (Simões and Riley, 2018). Within the subepicardial space, tissue-resident macrophages co-exist with both coronary and lymphatic endothelium. Our study attributes novel function to these resident macrophages in regulating the onset of cardiac lymphatic growth and patterning, along the base-apex axis and laterally to cover both dorsal and ventral surfaces of the developing heart. Tissue-resident macrophages were observed proximal and in direct contact with lymphatic vessels, where they accumulated at branch points. Moreover, in two independent mutant mouse models, *Pu.1^−/−^* and *Cx3cr1^CreER/+^;R26R-DTA*, macrophage depletion resulted in a truncated and mis-patterned lymphatic plexus, suggesting an essential role in modulating the extension and branching of the lymphatic endothelium (Figs 3 and 5). With regards to non-yolk sac-derived macrophages (i.e. foetal liver), these only colonize the developing heart at a timepoint when the cardiac lymphatic network is already well-established (from E14.5 onwards), and, therefore, we can discount these cells from being relevant for the onset of cardiac lymphatic expansion, but we cannot rule out a role at later developmental stages (e.g. maintenance versus remodelling of the vascular network).

A previous study reported an analogous role for embryonic macrophages in the remodelling of the primitive coronary blood plexus (Leid et al., 2016). Here, the authors revealed that resident macrophages control the selective expansion of perfused blood vessels, and that an absence of macrophages resulted in hyper-branching of the developing coronaries (Leid et al., 2016). In contrast, we observed a transient reduction in vessel growth and branching (Fig. 4), and a mild, but reproducible, mis-patterning of the coronary vessels following macrophage depletion in *Pu.1^−/−^* and *Cx3cr1^CreER/+^;R26R-DTA* mutant mice (Figs 4 and 6). Our findings are supported by studies documenting reduced vessel branching in alternative vascular beds of mice lacking embryonic macrophages, such as the developing hindbrain and postnatal retina (Fantin et al., 2010). Thus, the differences in severity of phenotype between our analyses and those of Leid and colleagues could be due to distinct mouse models used and/or approaches employed to characterize the coronary vasculature [whole-mount heart immunostaining in this study versus analyses of cryosections in previous work (Leid et al., 2016)].

At a molecular level, the remodelling of the lymphatic vasculature could arise from macrophage phagocytic activity, release of soluble cytokines or cell-cell interactions. Tissue-resident macrophages have been reported to control various processes during vessel development, in a variety of blood and lymphatic vascular beds, mediated by secretion of trophic factors. These include regression of the transient hyaloid endothelium by inducing WNT7B-driven cell death (Lobov et al., 2005), inhibition of branch formation in the developing lymphatic system in the diaphragm through secretion of an elusive factor (Ochsenbein et al., 2016), controlling LEC proliferation in dermal lymphatics (Gordon et al., 2010) and mediating coronary blood plexus remodelling through selective expansion of the perfused vasculature via insulin-like growth factor (IGF) signalling (Leid et al., 2016). In contrast, our findings in the developing mouse heart and *in vitro* models of human lymphatic capillary tube formation and sprouting (Figs 3, 5 and 7) favour a mechanism mediated by direct macrophage-endothelial cell physical contact/adhesion (Fig. 1), similar to the role of macrophages in the developing hindbrain and retinal vasculature (Fantin et al., 2010). In this regard, a requirement for macrophage HA in lymphatic sprouting was identified (Fig. 7). HA is a key structural component of the vertebrate extracellular matrix and has recently been implicated in the postnatal development and response to adult injury (inflammation driven) of murine corneal lymphangiogenesis (Sun et al., 2019), a process that also depends on the presence of macrophages (Maruyama et al., 2012). Hyaluronan levels are determined, partly by three hyaluronan synthases (HAS1-HA3) and three hyaluronidases (HYAL1-HYAL3) (Triggs-Raine and Natowicz, 2015). HA is also known to interact with different cell surface receptors, including LYVE1, CD44 and HMMR/CD168, to directly influence endothelial cell motility, proliferation and survival, although the role of such receptors in the regulation of hyaluronan levels remains elusive (Savani et al., 2001; Triggs-Raine and Natowicz, 2015). As such, functional redundancy of different HA-binding proteins likely contributes to the lack of a developmental (lymphatic) phenotype in *Lyve1* and *Cd44* single and compound knockout mice (Gale et al., 2007; Luong et al., 2009). Conversely, genetic disruption of hyaluronan synthesis either abrogated normal cardiac morphogenesis, leading to mid-gestation lethality, or impaired the response of cardiac macrophages to ischemia reperfusion injury, resulting in poor macrophage survival and functional cardiac output (Camenisch et al., 2000; Petz et al., 2019). Thus, further studies are needed to dissect out the HA/HA-binding receptor axis required for macrophage-induced lymphatic sprouting. In particular, it will be important to investigate the requirement for HA-mediated signalling in macrophages residing in the subepicardial compartment during the early stages of cardiac lymphatic growth. Likewise, an indirect effect, via the release of a soluble growth factor(s) by tissue-resident macrophages cannot be categorically ruled out at this stage, albeit we can discount VEGFC given its undetectable expression by macrophages in the developing heart (Fig. S5). In addition, future studies investigating subepicardial resident-macrophages should focus on expanding the phenotypic characterization of these cells, e.g. via the use of unbiased single cell RNA-sequencing (scRNA-seq) platforms followed by careful examination/validation *in situ* (mapped against lymphatic expansion) and selective cell targeting (based on their marker profile).

We assign a novel function to the yolk sac-derived tissue-resident macrophage lineage, against a back-drop of a rapidly evolving field that attributes ever increasing plasticity, in terms of cell fate and signalling, to these essential resident lineages. This is complemented by insights into our fundamental understanding of how discrete organ-based vascular beds are formed and the degree of heterogeneity in terms of cellular contributions to organ-specific endothelium. Live imaging in the adult mouse and zebrafish has demonstrated that inflammatory macrophages are required for orchestration of skin wound neoangiogenesis (Gurevich et al., 2018). Likewise, macrophages of diverse phenotypes support vascularization of 3D human engineered tissues (Graney et al., 2020). Moreover, the maintenance of the mouse choroidal vascular network, as well as lymphatic vessels in the cornea, is dependent on the presence of macrophages, with decreased macrophage number and activation leading to reduced lymphatic vessel formation and contributing to impaired diabetic wound healing in a mouse model of corneal wound healing (Maruyama et al., 2007, 2012; Yang et al., 2020). Importantly, the close interaction between cardiac lymphatics and macrophages during development also appears to manifest in the postnatal heart via infiltrating inflammatory macrophages and injury-activated lymphangiogenesis following myocardial infarction (Klotz et al., 2015; Vieira et al., 2018). The functional role for macrophages in directly regulating lymphatic growth and remodelling during development we report herein may, therefore, manifest in response to injury in the adult setting. Taken together, this further strengthens the rationale for extrapolating our developmental studies to therapeutically target macrophage-lymphatic responses during postnatal cardiovascular disease/injury.

## MATERIAL AND METHODS

### Mouse lines

Genetically modified mouse lines used in the study were kept in a pure C57BL/6 genetic background and are listed in Table S1. *Cx3cr1^GFP^* knock-in mice and *hCD68-GFP* transgenic mice were crossed with C57BL/6 to generate *Cx3cr1^GFP/+^* and *hCD68-GFP^+^* samples, respectively. *Pu.1^+/−^* mice were intercrossed to generate *Pu.1^−/−^* specimens. *Csf1r-CreER* transgenic mice, *Cx3cr1^CreER/+^* knock-in mice and *Flt3^CreERT2/+^* mice were crossed with *R26R-tdTomato* or *R26R-DTA* reporter strains to generate *Csf1r-CreER;tdTomato*, *Cx3cr1^CreER/+^;R26R-tdTomato*, *Cx3cr1^CreER/+^;R26R-DTA* and *Flt3^CreERT2/+^;tdTomato* mice. Both males and females were used in the study. For timed-mating experiments, 8- to 16-week-old mice were set up overnight and females checked for vaginal plugs the following morning; the date of a vaginal plug was set as embryonic day (E) 0.5. For tamoxifen-dependent tissue-specific gene activation, a 75 mg/kg dose of tamoxifen (Sigma-Aldrich) was administered to pregnant dams, intraperitoneally. Mice were housed and maintained in a controlled environment by the University of Oxford Biomedical Services. All animal experiments were carried out according to the UK Home Office project license (PPL) 30/3155 and PPDE89C84 compliant with the UK animals (Scientific Procedures) Act 1986 and approved by the local Biological Services Ethical Review Process.

### Cell lines

Primary human dermal lymphatic endothelial cells (HDLEC) isolated from the dermis of juvenile foreskin (PromoCell) were cultured in the presence of endothelial cell growth medium (ECGM)-MV2 (PromoCell), according to the manufacturer’ instructions. Human iPS-derived macrophages constitutively expressing RFP (Haenseler et al., 2017) were obtained from the James Martin Stem Cell Facility (Sir William Dunn School of Pathology, University of Oxford, UK). The human iPS cell line was derived previously from dermal fibroblasts of a healthy donor that had given signed informed consent for the derivation of iPS cells [Ethics Committee: National Health Service, Health Research Authority, NRES Committee South Central, Berkshire, UK (REC 10/H0505/71)]. iPS-macrophages were cultured in macrophage medium composed of X-VIVO 15 (Lonza), GlutaMax (Invitrogen) and M-CSF (Invitrogen), as previously described (van Wilgenburg et al., 2013). Cell lines were maintained in a humidified atmosphere of 5% CO_2_ at 37°C. Of note, results arising from co-cultures of human LECs (HDLECs) and iPS-derived macrophages expressing RFP (Fig. 7) were validated by using the alternative primary human dermal microvascular endothelial cells (HMVEC-dAD), derived from an adult donor (Lonza), and human wild-type (WT) macrophages (no reporter) obtained from the James Martin Stem Cell Facility (Sir William Dunn School of Pathology, University of Oxford, UK). The latter were derived from the human iPS cell line SFC856-03-04.

### Sample preparation for immunostaining

Embryos were harvested at the required embryonic stage, placed in ice-cold PBS (Sigma-Aldrich) and the amniotic sac was removed. The heart was micro-dissected from the embryo for immunostaining experiments using fine forceps. Dissected hearts were fixed for 6 h in 2% PFA/PBS at room temperature, permeabilized in 0.3% Triton X-100/PBS (twice, 10 min each) and then blocked in 1% BSA, 0.3% Triton X-100 in PBS for at least 2 h. Samples from fluorescent reporter lines (e.g. *Cx3cr1^GFP^* and *CD68-GFP*) were protected from light throughout this procedure. Samples were incubated with primary antibodies (diluted in block; listed in Table S1) overnight at 4°C, then washed three times for at least 1 h each in 0.3% Triton X-100/PBS. Samples were incubated with Alexa Fluor-conjugated secondary antibodies (diluted in PBS; Invitrogen) overnight at 4°C, protected from light, then rinsed three times for at least 10 min each with PBS. The hearts were then orientated according to dorsal-ventral surface aspect and mounted in 50% glycerol/PBS in glass-bottomed dishes (Mattek). Imaging was performed using an Olympus FV 1000 or Zeiss LSM 780 scanning confocal microscope. Tiled images were acquired using a 10× objective to include an overview of whole hearts and at least three different fields were captured at higher magnification (20× objective) per heart surface aspect (dorsal vs ventral) per sample. Images were digitally captured and processed using Zen and FIJI-ImageJ software. Analysis of total vessel length and junction number calculation were performed using AngioTool software that enables automated assessment of the entire vascular network covering the subepicardial surface (Zudaire et al., 2011). Briefly, the quantitative analyses of vascular networks (lymphatic and blood vessels) were carried out by tracing vessels only using ImageJ and then analysing the traced networks on AngioTool. Of note, throughout this study the cardiac lymphatic vasculature was labelled using (at least) dual combinations of antibodies against LECs, such as LYVE-1/VEGFR3 or LYVE-1/PROX1. This strategy allowed combination with a third antibody to label coronary vessels (e.g. EMCN), thereby maximizing the information extracted/sample and, more importantly, excluding a possible bias when tracing the vessels arising from LYVE-1 labelling of both lymphatic endothelium and tissue-resident macrophages. At least two independent litters and three embryos were used to generate the data discussed here, with representative images being shown in main figures and supplementary figures.

### Preparation of single cell suspensions from the heart

Foetal hearts harvested for flow cytometry studies were isolated, placed in ice-cold HBSS (Life Technologies), finely minced into small pieces and digested with collagenase type II (Worthington Laboratories) solution (containing 500 units/ml HBSS) at 37°C for 30 min with agitation at 180 rpm. Dissociated samples were then passed into a 50 ml conical tube (Corning) through a 70 µm cell strainer, then rinsed with 3 ml ice-cold HBSS and transferred back to a 15 ml conical tube. Samples were spun for 7 min at 350 ***g*** at 4°C, and the supernatant carefully discarded. Cell pellets were resuspended in 5 ml of red blood cell (RBC) lysis buffer (BioLegend) and incubated at room temperature for 10 min, followed by a repeat centrifugation at 350 ***g*** at 4°C for 7 min. The RBC lysis buffer was removed and cells were resuspended in 2% FBS/PBS. Isolated single cardiac cells were stained and subjected to flow cytometry analyses.

### Flow cytometry

Using 7-AAD exclusion (BD Pharmingen), only live cells were analysed. All antibodies used for flow cytometry are listed in Table S1. Flow cytometric analyses were performed using FACSAria III flow cytometer (BD Biosciences) and FlowJo software (LLC). Samples resuspended in 2% FBS/PBS solution were blocked with mouse Fc Block (Miltenyi Biotec) for 5 min on ice, followed by labelling for 20 min at room temperature with each antibody combination.

### Time-lapse tube formation assay

384-well plates (PerkinElmer) were coated with growth factor-reduced Matrigel (Corning) and incubated for 30 min at 37°C. ECGM-MV2 containing 4500 HDLECs (25 μl per well) was added and incubated for 60 min at 37°C. Macrophage media containing 2250 human iPS-derived macrophages-RFP was then added (25 μl per well). For time-lapse imaging acquisition, an Evos FL Auto Cell Imaging System (Thermo Fisher Scientific) was used and one image was taken every 8 min for 15 h. At the end of image acquisition, cells were fixed with 4% PFA solution (Santa Cruz Biotechnology) for 30 min and washed with PBS for further analysis (e.g. immunostaining).

### Immunostaining of HDLEC-macrophage co-cultures

HDLEC-like tubes were permeabilized with 0.1% Triton X-100/PBS and then incubated with a staining solution containing Alexa Fluor 568 Phalloidin and DAPI (both Invitrogen). Immunofluorescence staining was performed using an antibody against human CD68 (Abcam; see Table S1).

### iPS-derived macrophage transfection

Macrophages were transfected in a 24-well plate as previously described (Troegeler et al., 2014). Macrophages were washed twice with warm complete XVIVO in order to remove floating cells. Fresh complete XVIVO was added (250 μl per well) and cells kept at 37°C and 5% CO_2_. siRNA (3.75 μl of 20 μM; Thermo Fisher Scientific), 110.25 μl XVIVO depleted (without MCSF) and 11 μl HiPerfect (Qiagen) were gently mixed in a tube and incubated at room temperature for 15-20 min. 125 μl of the mix was then added in each well drop by drop. The plate was gently rocked to ensure even distribution of the mix. After 6 h, 0.5 ml of Complete XVIVO was added and the cells were harvested 3 days later for RNA extraction and/or experimental procedures.

### HAase treatment and immunostaining

Macrophages were seeded on coverslips and treated with 15 U/ml or 30 U/ml hyaluronidase (HAase; Sigma-Aldrich) 2 h before the experiment. For staining, cells were fixed with 4% PFA and permeabilized in 0.2% Triton X-100/PBS for 10 min and then blocked in 1% BSA, 0.1% Triton X-100 in PBS for at least 2 h. Macrophages were incubated with 3 μg/ml biotinylated hyaluronan-binding protein (Amsbio) overnight at 4°C, then washed three times with PBS. Samples were incubated with Alexa Fluor 488 Streptavidin (diluted in PBS; Biolegend) overnight at 4°C, protected from light, then rinsed three times for at least 10 min each with PBS. Coverslip were then mounted on slides using Vectashield+DAPI (Vector Laboratories).

### Microbeads capillary sprouting assay

The capillary sprouting assay was performed as previously described previously (Schulz et al., 2012). HDLECs were incubated in a 15 ml conical tube in presence of cytodex 3 beads (Sigma-Aldrich) at a ratio of 400 cells per bead in EGM-2MV medium (Lonza) for 4 h at 37°C with shaking every 20 min. Beads were then incubated in a cell flask at 37°C for 48 h in EGM-2MV medium. The HDLEC-coated beads were subsequently collected and labelled with 2 μM Cell Tracker Green dye (Invitrogen) for 30 min, embedded in 1 mg/ml Type I collagen hydrogel (Advanced Bio-Matrix) containing 2 μM D-erythro-sphingosine-1-phosphate (Avanti Polar Lipids), and cultured in black clear-bottom 96-well plates (Perkin-Elmer) in the presence or absence of 10,000 human iPS-derived macrophages-RFP per well. After 60 min of incubation, a 1:1 mix containing EGM2-Incomplete (EGM2 media without supplementation with EGF, FGF2 and VEGF-A; Lonza) and macrophage media (v/v) was added and the beads incubated for 2 days at 37°C. The beads were then fixed with 4% PFA for 30 min and washed twice with PBS. Each well was imaged with a Zeiss LSM 780 scanning confocal microscope and the number of sprouts per bead calculated.

### Spheroids sprouting assay

Spheroids of 400 HDLECs were generated using a 24-well plate Aggrewell 400 (Stemcells Technologies) using EGM2-Incomplete. The following day, spheroids were collected and embedded in 1 mg/ml Type I collagen hydrogel and cultured in black clear-bottom 384-well plates (Perkin-Elmer) in the presence or absence of 2500 human iPS-derived macrophages-RFP per well. After 60 min of incubation, a 1:1 mix containing EGM2-Incomplete (Lonza) and macrophage media (v/v) was added, and the spheroids incubated for 2 days at 37°C. The spheroids were then fixed with 2% PFA for 30 min, washed twice with PBS and stained with Alexa Fluor 488 phalloidin (Invitrogen). Each well was imaged with an automated cell imaging system (Pico microscope, Molecular Devices) and the number of sprouts per spheroids calculated.

### RNA extraction and qRT-PCR

Total RNA was extracted using Trizol Reagent (Invitrogen). Reverse-transcription (RT) was performed using SuperScript(R) III Reverse Transcriptase (Invitrogen), as recommended by the manufacturer. Primer sequences (Invitrogen) used were: *36B4*_F, CTACAACCCTGAAGAAGTGCTTG; *36B4*_R, CAATCTGCAGACAGACACTGG; *CSF1R*_F, CCTCACTGGACCCTGTACTC; *CSF1R*_R, GGAAGGTAGCGTTGTTGGTG; *CX3CR1*_F, ACCAACTCTTGCAGGTCTC; *CX3CR1*_R, TGTCAGCACCACAACTTGG; *ITGB2/CD18*_F, GTCTTCCTGGATCACAACGC; *ITGB2/CD18*_R, CAAACGACTGCTCCTGGATG; *ITGAM/CD11b*_F, CAGTGAGAAATCCCGCCAAG; *ITGAM/CD11b*_R, CCGAAGCTGGTTCTGAATGG; *VEGFC*_F, GGAGGCTGGCAACATAACAG; *VEGFC*_R, TTTGTCGCGACTCCAAACTC; *LYVE1*_F, CTGGGTTGGAGATGGATTCG; *LYVE1*_R, TCAGGACACCCACCCCATTT; *CD44_*F, AAGTGGACTCAACGGAGAGG; *CD44_*R, GTCCACATTCTGCAGGTTCC; *HMMR/CD168*_F, GGCTAAGCAAGAAGGCATGG; and *HMMR/CD168*_R, TCCCTCCAGTTGGGCTATTT.

Real-time polymerase chain reaction (PCR) assays were run on a ViiA 7 step real-time PCR machine (Applied-Biosystems). Normalization was performed using *36B4* as a reference gene. Quantification was performed using the comparative Ct method.

### scRNA-seq and analysis

E7.75_E8.25_E9.25 10x Chromium data (GSE126128) were downloaded from UCSC Cell Browser as raw UMI count matrix (de Soysa et al., 2019). Only the E9.25 datasets were selected for further analysis. E10.5 heart 10x Chromium data were downloaded as raw counts from GEO (GSE131181) (Hill et al., 2019). All scRNA-seq datasets were analysed using Seurat (Butler et al., 2018; Stuart et al., 2019) in R as follows: individual replicates were integrated using SCTransform method; principal component analysis was used to define the cell clusters, which were visualized with the UMAP method (Becht et al., 2018; Butler et al., 2018).

### Statistical analysis

All data are presented as mean±s.e.m. Statistical analysis was performed on GraphPad Prism 8 software. The statistical significance between two groups was determined using an unpaired two-tailed Student's *t*-test; these included a *F*-test to confirm the two groups had equal variances. Among three or more groups (e.g. data shown in Fig. 1A), one-way analysis of variance (ANOVA) followed up by Tukey's multiple comparison test was used for comparisons. *P*≤0.05 was considered statistically significant.

## Supplementary Material

Supplementary information

Reviewer comments
