# Supplementary material for: Tissue-resident macrophages regulate lymphatic vessel growth and patterning in the developing heart
Source: Development. 2021 Feb 3;148(3):dev194563. doi: 10.1242/dev.194563 (PMC7875498; doi:10.1242/dev.194563)
Supplement: Supplementary information [file develop-148-194563-s1.pdf]

SUPPLEMENTARY FIGURE LEGENDS

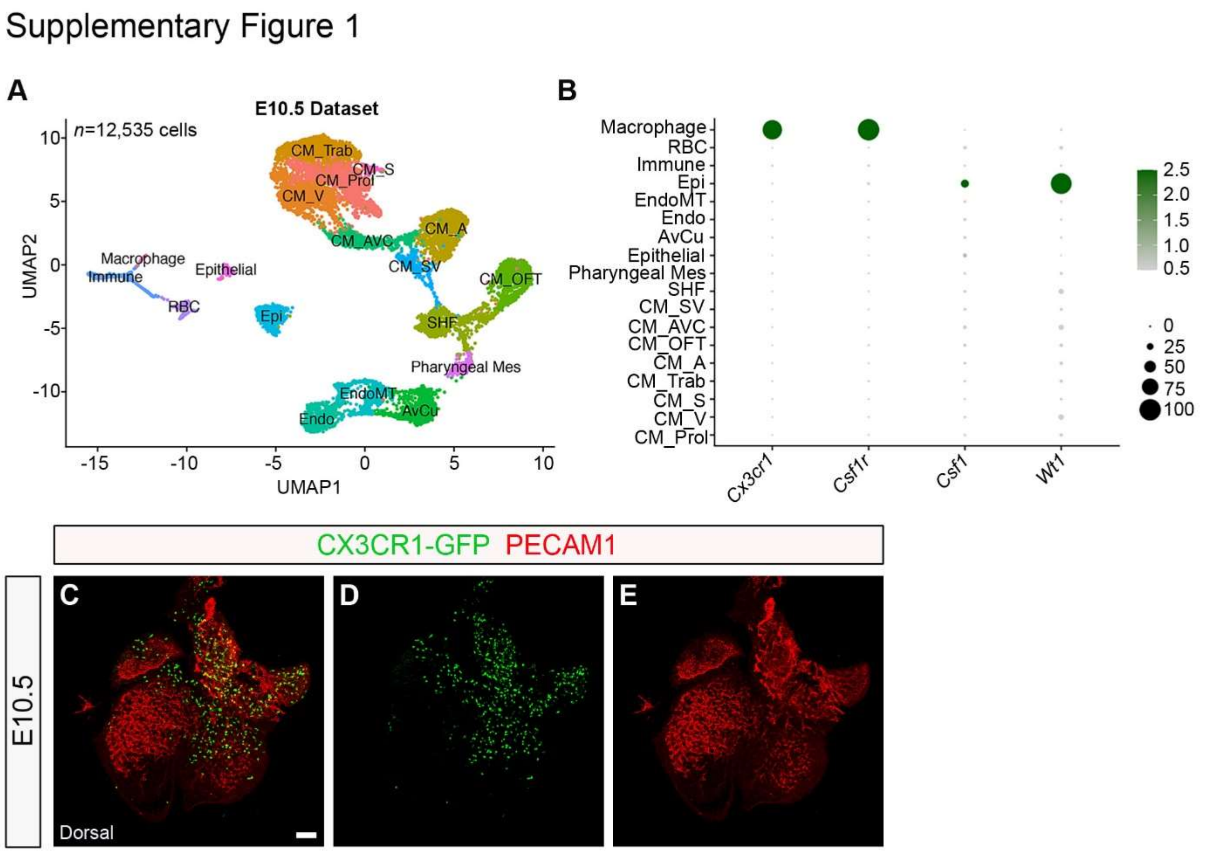

Fig. S1. Tissue-resident macrophages are present in the developing murine heart at E10.5. (A) UMAP plot demonstrating the different major clusters in the E10.5 heart scRNA-seq dataset (total 12,535 cells, *n* = 3 batches). (B) Dot plot showing proportion of cells in each cluster expressing selected genes. Dot size represents percentage of cells expressing, and color scale indicates average expression level. (C-E) Representative whole-mount immunostaining for GFP (green), and PECAM-1 (red) to visualize tissue-resident macrophages in the sinus venosus and ventricular surface of hearts-derived from *Cx3cr1*$^{GFP/+}$ embryos at E10.5. AvCu, atrioventricular cushion; CM_A, atrial cardiomyocytes; CM_AVC, atrioventricular canal cardiomyocytes; CM_OFT, cardiomyocytes of outflow tract; CM_Prol, proliferative cardiomyocytes; CM_S, septal cardiomyocytes; CM_SV, sinus venosus cardiomyocytes; CM_Trab, trabecular cardiomyocytes; CM_V, ventricular cardiomyocytes; Endo, endocardial cells; EndoMT, endocardial epithelial-to-mesenchymal transition; Epi, epithelial cells; Mes, mesenchyme; RBC, red blood cells; SHF, second heart field. Scale bar 100 μm

Development • Supplementary information

Supplementary Figure 2

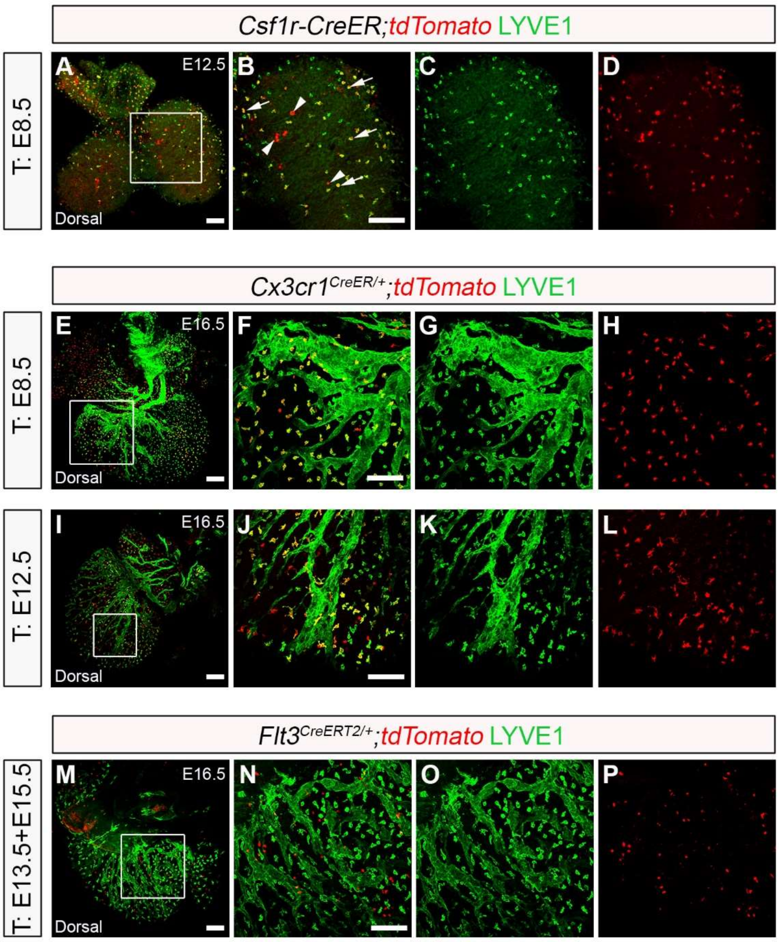

Fig. S2. Characterization of *Csf1r-CreER*, *Cx3cr1*^{CreER} and *Flt3*^{CreERT2} lineage tracing mouse lines. (A-D) Genetic lineage-tracing based on the activity of the *Csf1r-CreER;tdTomato* transgene induced by tamoxifen administration at embryonic day (E)8.5. Whole-hearts were analyzed for Tomato (red) and LYVE-1 (green) expression at E12.5. (B-D) Magnified views of box shown in (A). White arrows indicate *Csf1r*$^{+}$ lineage-derived cells expressing the macrophage marker LYVE-1; white arrowheads indicate *Csf1r*$^{+}$ lineage-derived cells lacking macrophage marker expression residing in the developing heart at E12.5. (E-L) Genetic lineage-tracing based on the activity of the *Cx3cr1*$^{CreER/+}$*;tdTomato* reporter induced by tamoxifen administration at E8.5 (E-H) or E12.5 (I-L). Whole-hearts were analyzed for Tomato (red) and LYVE-1 (green) expression at E16.5. (F-H) Magnified views of box shown in (E). (J-L) Magnified views of box shown in (I). (M-P) Genetic lineage-tracing based on the activity of the *Flt3*$^{CreERT2/+}$*;tdTomato* reporter induced by combined tamoxifen administration at E13.5 and E15.5. Whole-hearts were analyzed for Tomato (red) and LYVE-1 (green) expression at E16.5. (N-P) Magnified views of box shown in (M). *n* = 3 - 6 hearts per group from at least three independent litters. All scale bars100 µm

Development • Supplementary information

Supplementary Figure 3

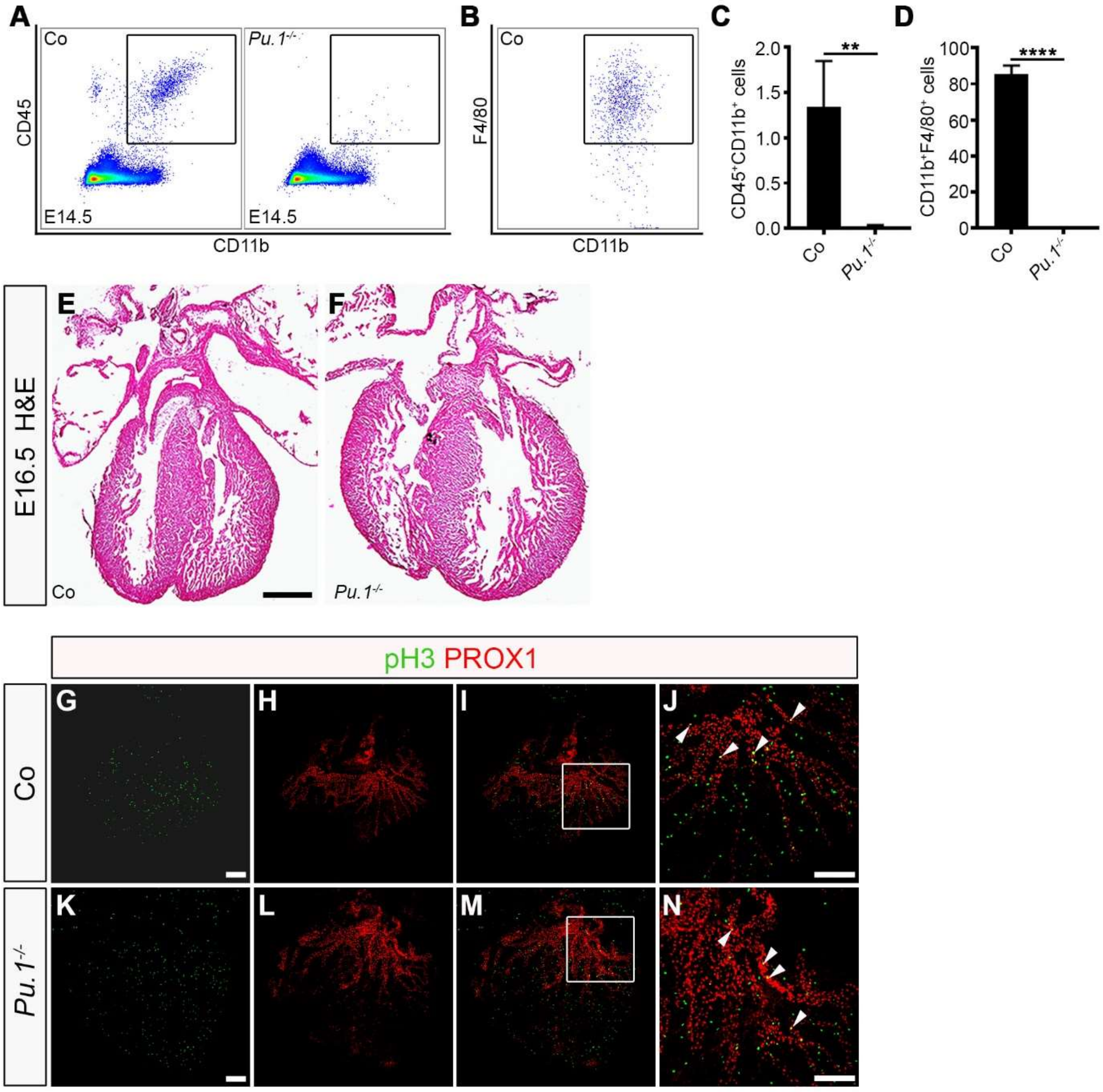

Fig. S3. Loss of macrophages is not associated with hyperproliferation of the cardiac lymphatic endothelium. (A,B) Representative flow cytometry histograms of cells isolated from control (Co) and *Pu.1*$^{-/-}$ hearts at E14.5 labeled against CD45 and CD11b (A) or F4/80 and CD11b markers (B). (C,D) Quantification of the myeloid (C), defined as CD45^{+}CD11b^{+}, and macrophage populations, defined as CD45^{+}CD11b^{+}F4/80^{+} (D) residing in Co versus *Pu.1*$^{-/-}$ hearts at E14.5. Data represent mean ± SEM; $n = 4$ hearts per group from three independent litters. Significant differences (p values) were calculated using an unpaired, two-tailed Student's t-test (**$p \leq 0.01$). (E,F) Histological characterization of control and *Pu.1*$^{-/-}$ hearts at embryonic day (E) 16.5 using Hematoxylin & Eosin staining. (G-N) Whole-mount immunofluorescent staining for phospho-histone H3 (pH3; green) and PROX1 (red) in control (Co; G-J) and *Pu.1*$^{-/-}$ hearts (K-N) at E16.5. All scale bars 100 µm, except E 1mm.

Development • Supplementary information

Supplementary Figure 4

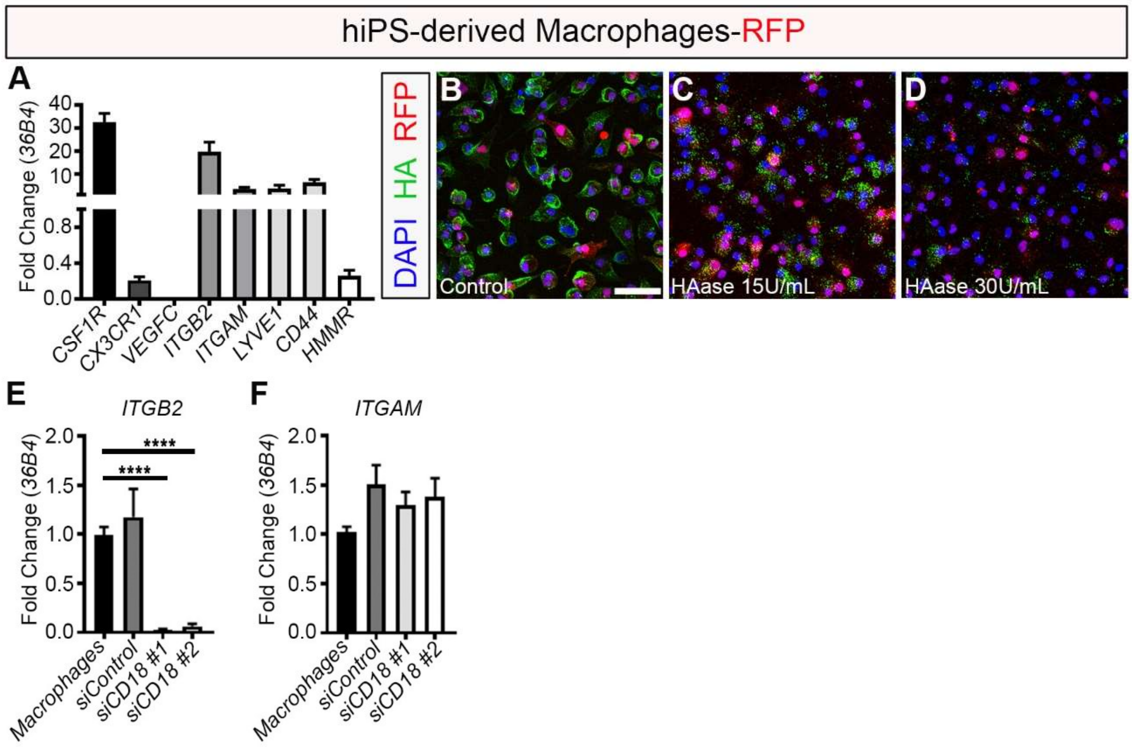

Fig. S4. Human induced pluripotent stem cell (hiPS)-derived macrophages display HA on their cell surface, express HA-binding proteins and adhesion receptor CD18/CD11b but lack expression of *VEGFC*. (A) Marker expression analysis by qRT-PCR using RNA isolated from hiPS-macrophages. Data presented as mean ± SEM; *n* = 6 independent experiments, with three replicates/experiment. (B-D) Representative HA staining (green) on hiPSC-derived macrophages-RFP (red) under control conditions (B) or following incubation with HAase 15 U/mL (C) and HAase 30 U/mL (D). DAPI (blue) labels the nuclear DNA. Note the loss of cell surface (glycocalyx)-associated HA following treatment with HAase 30 U/mL. (E) *ITGB2* and (F) *ITGAM* expression analysis by qRT-PCR using RNA isolated from control hiPS-macrophages (macrophages) or hiPS-macrophages treated with control siRNA or siRNA oligonucleotides against *ITGB2*/*CD18* (two independent siRNA sequences were used; siCD18 #1 and siCD18 #2). Data presented as mean ± SEM; *n* = 6 independent experiments, with three replicates/experiment. Significant differences (*p* values) were calculated using one-way ANOVA followed up by the Tukey's multiple comparison test (****$p \leq 0.0001$). All scale bars 200 μm.

Supplementary Figure 5

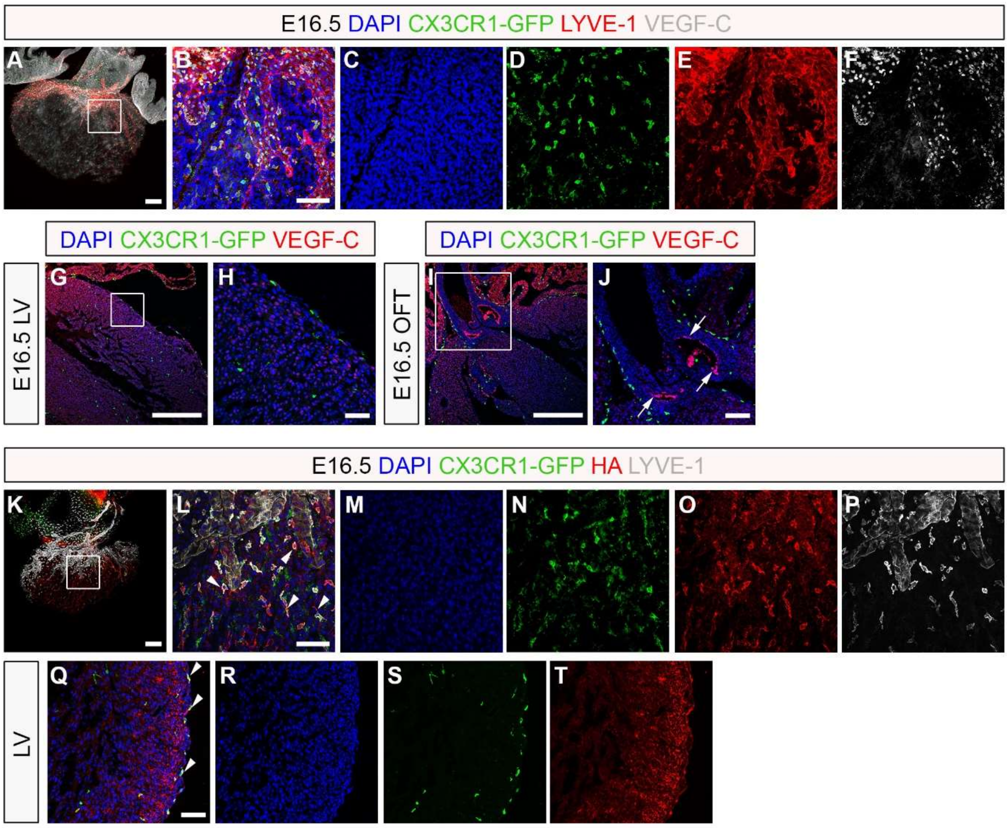

Fig. S5. Tissue-resident macrophages in the developing mouse heart display hyaluronan on their cell surface and lack expression of VEGF-C. (A-F) Whole-hearts from *Cx3cr1*^{GFP} embryos were analyzed for GFP (green), LYVE-1 (red) and VEGF-C (white) expression at E16.5. (B-F) Magnified views of box shown in (A). Note VEGF-C co-localization with LYVE-1 on lymphatic endothelium, but not on LYVE-1 and GFP co-expressing macrophages. (G-J) GFP (green) and VEGF-C (red) immunostaining of tissue sections derived from E16.5 hearts documenting undetectable expression of VEGF-C in CX3CR1-GFP^{+} ventricular/subepicardial macrophages (G,H) and enrichment for VEGF-C in the coronary stems (white arrows; I, J). (H,J) Magnified views of boxes shown in (G,I). (K-P) Whole-hearts from *Cx3cr1*^{GFP} embryos were analyzed for GFP (green), HA (red) and LYVE-1 (white) expression at E16.5. (L-P) Magnified views of box shown in (K). Note HA colocalization with GFP and LYVE-1 in tissue-resident macrophages located in the subepicardial space, contiguous to the expanding lymphatic vasculature (white arrowheads in L). (Q-T) GFP (green), HA (red) and LYVE-1 (white) immunostaining of tissue sections derived from E16.5 hearts indicating expression of HA in CX3CR1-GFP^{+} subepicardial macrophages (white arrowheads in Q). DAPI (blue) labels nuclear DNA. LV, left ventricle; OFT, outflow tract. All scale bars100 µm, except H 25 µm and J,Q 50 µm.

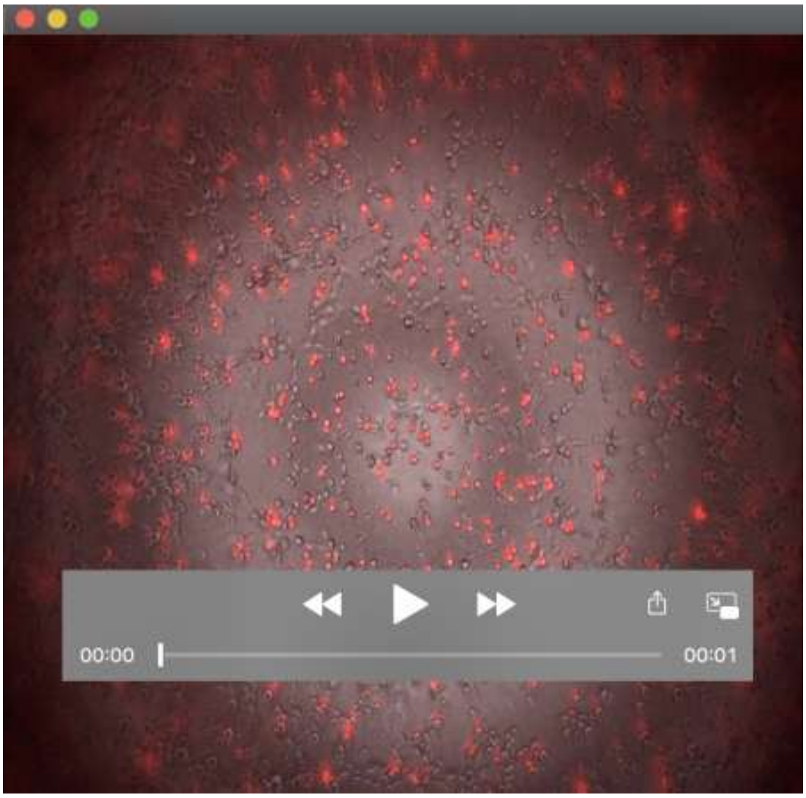

Movie S1. Time-lapse imaging of tube formation in co-cultures of human primary lymphatic endothelial cells (phase contrast) and human-iPS-derived macrophages expressing RFP.

TABLE S1. Key resources table.

REAGENT or RESOURCE	SOURCE	IDENTIFIER
**Antibodies**		
Rat monoclonal anti-mouse EMCN (clone V.5C7)	Santa Cruz Biotech (1:50)	Cat# sc-53941
Rabbit polyclonal anti-mouse LYVE-1	AngioBio (1:100)	Cat# 11-034
Armenian Hamster monoclonal anti-mouse PECAM-1/CD31 (clone 2H8)	Abcam (1:100)	Cat# ab119341
Rat monoclonal anti-mouse LYVE-1 (clone ALY7)	Novus Biologicals (1:100)	Cat# NBP1-43411
Goat polyclonal anti-mouse VEGFR3/Flt4	R&D (1:100)	Cat# AF743
Goat polyclonal anti-human PROX1	R&D (1:100)	Cat# AF2727
Chicken polyclonal anti-GFP	Abcam (1:200)	Cat# ab13970
Rat monoclonal anti-mouse CD68 (clone FA-11)	Bio-Rad (1:100)	Cat# MCA1957
Rabbit anti-VEGF-C	Abcam (1:100)	Cat# ab9546
Alexa Fluor 647 Rat anti-mouse CD45 (clone 30-F11)	Biolegend (1:100)	Cat# 103124
FITC Rat anti-mouse CD11b (clone M1/70)	Biolegend (1:100)	Cat# 101206
PE Rat anti-mouse F4/80 (clone BM8)	Biolegend (1:100)	Cat# 123110
Rabbit polyclonal anti-mouse p-Histone H3 (Ser10)	Santa Cruz Biotech (1:100)	Cat# sc-8656-R
Rat monoclonal anti-mouse CD31 (clone MEC13.3)	BD Pharmingen (1:100)	Cat# 550274
Mouse monoclonal Anti-human CD68 (clone KP1)	Abcam (1:100)	Cat# 955
Alexa Fluor 568 Phalloidin	Invitrogen (1:100)	Cat# A12380
**Chemicals, Peptides, and Recombinant Proteins**		
Paraformaldehyde (PFA) solution 4% in PBS	Santa Cruz Biotech	Cat# 281692
Cytodex 3 microcarrier beads	Sigma-Aldrich	Cat# C3275
7-amino-actinomycin D (7-ADD)	BD Pharmingen	Cat# 559925
DAPI solution	Invitrogen	Cat# 62248
EGM-2MV medium	PromoCell	Cat# C-22022
EGM2-Incomplete medium (EGM2 medium not supplemented with FGF, VEGF-A and EGF)	Lonza	Cat# CC-3162
Red Blood Cell lysis buffer (10x)	Biolegend	Cat# 420301
Collagenase, Type II	Worthington	Cat# LS004180
CellTracker Green CMFDA Dye	Invitrogen	Cat# C2925
Human Type 1 Atelo-Collagen solution, 1 mg/ml	Advanced Bio-matrix	Cat# 5007-20ML
D-erythro-sphingosine-1-phosphate (d18:1)	Avanti Polar Lipids	Cat# 860492P
**Experimental Models: Cell Lines**		
Human: Primary dermal lymphatic endothelial cells (HDLEC)	PromoCell	Cat# C-12216
Human dermal microvascular endothelial cells - adult (HMVEC-dAD)	Lonza	Cat#CC-2543
Human: iPS-derived macrophages-RFP	James Martin Stem Cell Facility- University of Oxford	Haenseler W et al. (2017) *Stem cell Reports* 8:1727.

Development • Supplementary information

Human: wild-type (WT) macrophages (no reporter) derived from human iPS cell line SFC856-03-04	James Martin Stem Cell Facility- University of Oxford	N/A
**Experimental Models: Organisms/Strains**		
Mouse: *Cx3cr1*^{GFP}: *B6.129P2(Cg)-Cx3cr1*^{tm1Litt}*/J*	The Jackson Laboratory	JAX: 005582
Mouse: *Cx3cr1*^{CreER}: *B6.129P2(Cg)-Cx3cr1*$^{tm2.1(cre/ERT2)Litt}$*/WganJ*	The Jackson Laboratory	JAX: 021160
Mouse: *Csf1r-CreER: FVB-Tg(Csf1r-cre/Esr1*)1Jwp/J*	The Jackson Laboratory	JAX: 019098
Mouse: *R26R-DTA: Gt(ROSA)26Sor*$^{tm1(DTA)Jpmb}$*/J*	The Jackson Laboratory	JAX: 006331
Mouse: *R26R-tdTomato: B6;129S6-Gt(ROSA)26Sor*$^{tm14(CAG-tdTomato)Hze}$*/J*	The Jackson Laboratory	JAX: 007908
Mouse: *Flt3*^{CreERT2}	Sten Eirik Jacobsen's laboratory, Karolinska Institutet, Sweden	Benz et al. (2008) *J. Exp. Medicine* 205: 1187.
Mouse: *hCD68-GFP*	David R. Greaves' laboratory, University of Oxford, UK	Iqbal et al. (2014) *Blood* 124: e33
Mouse: *Pu.1*$^{-/-}$	Paul Martin's laboratory, University of Bristol, UK	McKercher et al. (1996) *EMBO J.* 15: 5647.
**Software and Algorithms**		
Fiji-Image J	NIH	N/A
Photoshop	Adobe	N/A
Zen	Zeiss	N/A
Prism 8	GraphPad	N/A
AngioTool	Zudaire et al. (2011) *PLoS One* 6:e27385	N/A
FlowJo	LLC	N/A

Development • Supplementary information
