# Supplementary material for: Tissue-resident macrophages regulate lymphatic vessel growth and patterning in the developing heart
Source: Development. 2021 Feb 3;148(3):dev194563. doi: 10.1242/dev.194563 (PMC7875498; doi:10.1242/dev.194563)
Supplement: Reviewer comments [file develop-148-194563_review_history.pdf]

Tissue-resident macrophages regulate lymphatic vessel growth and patterning in the developing heart

Thomas J. Cahill, Xin Sun, Christophe Ravaud, Cristina Villa del Campo, Konstantinos Klaourakis, Irina-Elena Lupu, Allegra M. Lord, Cathy Browne, Sten Eirik W. Jacobsen, David R. Greaves, David G. Jackson, Sally A. Cowley, William James, Robin P. Choudhury, Joaquim Miguel Vieira and Paul R. Riley
DOI: 10.1242/dev.194563

Editor: Benoit Bruneau

Review timeline

Original submission:	30 June 2020
Editorial decision:	3 August 2020
First revision received:	7 December 2020
Accepted:	26 December 2020

Original submission

First decision letter

MS ID#: DEVELOP/2020/194563

MS TITLE: Tissue-resident macrophages regulate lymphatic vessel growth and patterning in the developing heart

AUTHORS: Thomas J Cahill, Xin Sun, Christophe Ravaud, Cristina Villa del Campo, Konstantinos Klaourakis, Irina-Elena Lupu, Allegra M Lord, Cathy Browne, Sten Eirik W. Jacobsen, David R Greaves, David G Jackson, Sally A Cowley, William James, Robin P Choudhury, Joaquim Miguel Vieira, and Paul R. Riley

I have now received all the referees' reports on the above manuscript, and have reached a decision. The referees' comments are appended below, or you can access them online: please go to BenchPress and click on the 'Manuscripts with Decisions' queue in the Author Area.

As you will see, the referees express considerable interest in your work, but have some significant criticisms and recommend a substantial revision of your manuscript before we can consider publication. If you are able to revise the manuscript along the lines suggested, which may involve further experiments, I will be happy receive a revised version of the manuscript. Your revised paper will be re-reviewed by one or more of the original referees, and acceptance of your manuscript will depend on your addressing satisfactorily the reviewers' major concerns. Please also note that Development will normally permit only one round of major revision.

We are aware that you may currently be unable to access the lab to undertake experimental revisions. If it would be helpful, we encourage you to contact us to discuss your revision in greater detail. Please send us a point-by-point response indicating where you are able to address concerns raised (either experimentally or by changes to the text) and where you will not be able to do so within the normal timeframe of a revision. We will then provide further guidance. Please also note that we are happy to extend revision timeframes as necessary.

Please attend to all of the reviewers' comments and ensure that you clearly highlight all changes made in the revised manuscript. Please avoid using 'Tracked changes' in Word files as these are lost

© 2021. Published by The Company of Biologists under the terms of the Creative Commons Attribution License (https://creativecommons.org/licenses/by/4.0/).

in PDF conversion. I should be grateful if you would also provide a point-by-point response detailing how you have dealt with the points raised by the reviewers in the 'Response to Reviewers' box. If you do not agree with any of their criticisms or suggestions please explain clearly why this is so.

Reviewer 1

Advance summary and potential significance to field

In this manuscript the authors characterize the role of tissue resident macrophages in the developing of the lymphatic system in the hearth using lineage tracing and loss of function experiments in vivo. This is a novel function for resident embryonic macrophages of yolk sac origin. The insights into the remodelling role of tissue resident macrophages in the embryo could could be exploited to study this phenomenon in the adult during cardiovascular repair and disease.

Comments for the author

The work is very interesting even if I have some concerns regarding some tamoxifen inducing protocols and subsequent interpretation of the results.

1) This comment concerns the lineage tracing experiments in Figure 2, which is critical for the interpretation of further experiments. I am not convinced that the Csfr1-CreER and Cxrcr1-CreER alleles label completely non-overlapping sets of cells. In fact, Csfr1 EMP labelling obtained with 4OHT pulse at E8.5 can result in labelling of pre-macrophages as Csfr1+ EMPs acquire Cxrcr1 expression later in development (Stremmel et al., 2018). This is especially relevant in the induction approach used here, as Tamoxifen persists longer in circulation than 4OHT. This would also explain the very similar phenotypes obtained with cell ablation of the two subsets. It would be helpful to have a more comprehensive characterization of the labelled cell subsets in E16.5 hearts and at shorter time points after Tamoxifen pulse induction as well, ideally with a method enabling quantification and analysis of multiple markers (e.g. flow cytometry).

2) Related to the previous point. As in the genetic cell ablation studies in Figure 5 and 6 the Tamoxifen pulse is administered at a different time point to enable macrophage seeding of the heart (E12.5), it would be helpful to have an analogous qualitative and quantitative characterization of the cell subsets labelled with this induction strategy.

3) As the authors show that Flt3-derived cells are present in the E16.5 heart does the genetic cell ablation of Flt3+ cells result in any detectable phenotype?

4) What is the cellular mechanism by which lymphatic endothelial cells are lost in Pu.1 null and in macrophage-ablated hearts? It is shown that there is no difference in proliferation at E16.5. Have the authors checked an earlier time point (e.g. E14.5)? Are there signs of apoptosis?

Reviewer 2

Advance summary and potential significance to field

In this manuscript, Cahill and colleagues investigated lymphatic vessel patterning in the heart and studied the potential contribution of macrophages in this process. To do so they used 3 different genetic models to deplete macrophages and performed whole mount staining to evaluate and characterize the growing vasculature. Overall, the manuscript is well written and the scope is unambiguous, even if the contribution of macrophages in lymphangiogenesis has been widely studied and the data on hyaluronan-dependent action, although interesting are still too preliminary. In order to strengthen the conclusion and reach enough significance to the Development readership, I believe that several new key and control experiments would be required, especially the ones recommended below to refine the lineage tracing and depletion timing, as well as the quantification of the experiments already shown.

© 2021. Published by The Company of Biologists under the terms of the Creative Commons Attribution License (https://creativecommons.org/licenses/by/4.0/).

Comments for the author

Major concerns:
Figure 1 A. The authors show the total count of CX3CR1+F4/80+ macrophages which increases in time, suggesting active recruitment and/or proliferation of macrophages. Is the percentage of macrophages relative to total live cells remaining constant over the 3 time points, or does their presence become more dense at a certain stage?
To evaluate whether the increase in macrophage numbers is due to recruitment or/and macrophage proliferation the authors should perform an additional KI67 staining.
- Fig 1Q: 2 different symbols should be used for the 2 types of interactions. Actually it is not clear in the high mag showed in Fig1Q whether there is a branch or a leading edge. it seems rather an enlarged vessel segments. the authors should provide a better example.
- In the lines 206-208, the authors state that “tissue-resident macrophages colonize the embryonic heart prior to the formation of the main vascular networks and adopt a spatial distribution in close proximity to, and in contact with, the forming lymphatics.” A quantification of the spatial distribution of the macrophages and an analysis of their preferential positioning close to lymphatic vessels is missing to support this claim.
-line 255: the authors should show the data from a single injection (ie 13.5 and 15.5 separately) to understand when HSC start to effectively contribute macrophages to the heart. If it's just from E15.5, then HSC-derived macrophages would be unlikely to contribute to lymphatic vessel patterning.
I think the authors should provide this piece of information by performing a time course of the tamoxifen pulse with this line.
- The authors state that “no direct contribution to cardiac lymphatic endothelium was observed.”, however in Fig. 2J two double positive cells are visible, which might be on different planes but this cannot be excluded in the way the data are currently presented. Please explain adequately or show orthogonal views.
-Fig. 2I-L: macrophages traced with this line appear to be less close to lymphatic vessels compared to the previous 2 approaches. Could the authors quantify this? Moreover, for all three approaches, the authors should quantify the percentage of labelled macrophages over the total macrophage population.
- In Fig. 3, to exclude a possible bias in the quantification given by LYVE-1+ macrophages in close proximity to lymphatic vessels which are present in WT but absent in KO mice, a staining for another lymphatic vessel marker such as NRP2 would be required, otherwise provide in the materials and methods the quantification strategy that didn’t take into account LYVE1+ macrophages.
- In Fig. 3, I am not convinced that the length of the lymphatic vessel is reduced. The authors should clarify what they actually measured. In the example pictures, the vessels appear to reach a similar extension towards the apex in both the control and the mutant. Did the authors instead quantify the length of each vessel segment and plotted the sum? Information on how quantifications were made should be added also to the methods. The authors should rather find a way to quantify the most obvious phenotype at this stage, ie the increased caliber/diameter of the vessels.
- Please choose another representative picture in Fig. 3 for panel L and P since PU.1-/- heart seems to have more branch points and similar length to the wt control.
-line 283: in order to exclude developmental delay in the mutants, the authors should compare other morphological parameters, eg the heart or the embryo size, the toes/fingers or the whisker follicle development (see eg https://embryology.med.unsw.edu.au/embryology/index.php/Mouse_Stages). Is there any difference?
-Fig. S2E,F: the mutant heart shown appears much bigger, is this consistent.
If yes, please discuss. The authors should provide a quantification. See also comment above.
- In the lines 287-289, the authors state that coronary blood vessels growth and patterning were mildly affected, however, the reduction in vessel length and number of junctions (Fig. 4I and J) is similar to the one observed for lymphatic vessels.
- Fig. 4P and R shows a denser network of blood vessels in PU.1-/- mice please quantify vessels number or replace the pictures.
-line 294: the authors should mention that a similar experiment was done with op mice and compare it to theirs (Leid et al., 2016). Moreover, this figure is outside the scope of the manuscript but I agree it is important as control. The author should move it to supplementary.

© 2021. Published by The Company of Biologists under the terms of the Creative Commons Attribution License (https://creativecommons.org/licenses/by/4.0/).

- In lines 295-296 the authors refer to a paper previously published by Gordon et al. describing how skin macrophages are important to define dermal lymphatic vessels’ caliber. The authors should evaluate if they also observe a similar difference in the skin. This would corroborate their data.
-Fig. S2G-N: the authors should quantify this experiment before discussing differences compraed to other reports. Please provide it.
-line 308: in this case the authors should show the lineage tracing with R26R-tomato also when pulsed at E12.5, especially as the number of macrophages in fig 5 does not seem much reduced. It could well be that at this stage the authors are lineage tracing and therefore depleting a higher than expected number of Csf1r+ EMP-derived LECs.
The authors should instead also provide the pulse at 8.5 as it will have more chances to affect a larger number of macrophages and they have already shown with the lineage tracing that targeting of Csf1r+ EMP-derived LECs is minimal. This approach will still be different from the pu.1 experiment which is constitutive and where also non myeloid cells (eg B cell progenitors) will be affected.
- lines 309-311: it is fundamental that the authors provide a quantification of the numebr of macrophages. From the pictures included in the figure it appears that the number of macrophages is not significantly affected in the mutants. How can the lymphatic phenotype be explained then if there is no significant reduction in macrophages? is the number of PROX1 nuclei any different?
-line 329: Also in this case it is fundamental that the authors prove with a lineage tracing with R26Rtomato pulsed at E12.5 that the only cells labelled are macrophages and quantify the percentage over the total macrophage population.
-line 333: it is fundamental that the authors provide a quantification of the numebr of macrophages.
-line 339: in order to suggest that, the authors should verify that HSC-derived monocytes/macrophages in the heart are Cx3cr1 positive or not either by antibody staining/FACS or by crossing the Cx3cr1 gfp reporter to the Flt3-creERT2;R26Rtomato. Moreover, the paper seems to lack the corresponding experiment of depleting HSC-derived macrophages with Flt3-Cre;R26R-DTA. To get a clear conclusion on what subpopulation of macrophages is required for heart lymphangiogenesis (YS-derived or HSC-derived) the authors should provide these data.
- Why the numbers between Figure 4 and Figure 5 are so different? Please explain adequately.
- Fig. 7 A-K: to demonstrate that the observation the authors made means something, the authors should compare the tube formation rate without and with the addition of the macrophages. In addition they should also use an independent cell line, such as fibroblast, to make sure that whatever difference is observed is macrophage-specific.
-Fig7L-P: no sprouting is visible in the no macrophage condition suggesting that it might not be a reliable model to study lymphatic sprouting in general. is sprouting occurring only in the presence of macrophages? if this is the case, it might still be used but it should be specified.
- Is the viability of HAase treated macrophages impaired?
- In vitro experiments are not focusing on heart LVs and/or macrophages.
Since, as the authors rightfully stated, both lymphatic vessels and macrophages from different locations possess peculiar characteristics, and that the findings from Gordon et al. on skin lymphatic vessels did not recapitulate what they saw in the heart, to strengthen their data it would be better to isolate both cell types from the embryonic heart and perform the same experiments. The authors should at least discuss this.

Minor:
-line 40: what coincides with lymphatic emergence? macrophage recruitment?
-line 97: not precise. the authors cite a review where this process is well explained. HSCs are not liver-derived but AGM-derived but then reside in the liver. Please rephrase. Eg "…including fetal liver-resident EMPs and HSCs"
-line 100: not correct. the initial pool of macrophages is mainly replenished by monocytes derived from liver-resident EMPs, see the Ginhoux&Guilliams 2016 review
- Please state the number of litters and animals used for all the figures presented. And fields analysed per sample in the materials and methods.
- Rat polyclonal anti-mouse CD31 (clone MEC13.3) BD Pharmingen. Was it the monoclonal antibody MEC13.3 or was it used a different polyclonal one?
- Only + SEM is shown in all figure legends and not ± SEM, please correct it. The SEM is in general really high, it would be better to see individual values.
-fig S1C: can the authors show across section (even virtual) through the specimen shown in S1C to better show where the macrophages reside within the cardiac tissue?

© 2021. Published by The Company of Biologists under the terms of the Creative Commons Attribution License (https://creativecommons.org/licenses/by/4.0/).

- Fig1. even though the images are beautiful, the authors should change the colour combination by showing LYVE1 in red and EMCN in blue (which is the least informative marker in this analysis), cause this will help the reader understand how many of the LYVE1+ macrophages are also CX3CR1+ and viceversa. Or add panels showing just the LYVE1 and CX3CR1 merge without using the white for LYVE1 to appreciate the colocalization. As it is shown at the moment it is difficult to appreciate which ones are the double positive macrophages. Especially at E12.5
- Fig 1J-M: the authors should add 3 different symbols to indicate examples in the referred panel of these 3 different types of association.
- line 198: CD68 gfp is shown only combined to VEGFR3. either rephrase or show gfp with lyve1 and prox1.
-line 203: the authors cannot say that those macrophages are promoting fusions as this is a static analysis. please rephrase.
-line 215: Csf1r is not expressed by the hemogenic endothelium but by the EMPs that emerge from such endothelium and also reside in the liver alongside dHSCs. Please correct.
-line 219: the authors should not use this abbreviation as it is confusing.
CreER is a completely different transgene which was the base for the generation of the mutated version CreERT2. Please stick to the original line name.
-line 221: most likely promoter. Please check the original paper
- line 232: most likely promoter. Please check the original paper
-line 240: if this is a knock in allele modification and not a transgene then the allele modification should be written in superscript without hyphen
-line 251: again modify line labelling according to the knockin allele
- In Fig. S2B a representative KO mouse FACS plot should be shown.
-line 343: the main difference with Pu.1 mutants is more likely to be the fact that the Cre approach is inducible and far way from targeting all the macrophages as in pu.1. The authors should mention this.
- In Fig. S3, not all macrophages are RFP positive. Are they selected by FACS before coculture?
- In line 409-412, the authors state that macrophages in the developing heart lack VEGF-C expression, however, with the type of analysis provided in Fig. S4 this claim is a bit too strong. In order to prove this, the authors should either perform a FACS sorting of macrophages and perform a qPCR or perform an RNA-FISH staining for VEGF-C.
Moreover, is VEGFC expression in the nucleus of LECs in panel F something expected? Any reference?
Please rephrase or provide such information.
- The authors claim that macrophages directly affect lymphatic vessel growth in the heart during development, however, besides blood vessels, they did not check and/or exclude the possible contribution of other cell types which could induce lymphangiogenesis, and might be affected by macrophage depletion. The authors should comment on this possibility in the discussion.

Reviewer 3

Advance summary and potential significance to field

The studies described in this paper characterize the role of tissue resident macrophages in the formation and remodeling of cardiac lymphatic vessels. The authors characterize malformations in cardiac lymphatics upon macrophage ablation in different mouse backgrounds during development. This work expands on previous publications, including from the same lab, analyzing the role of macrophages in lymphatic development. Here, the main focus is the contribution/role of organ-specific (heart)/tissue resident macrophages during the formation of cardiac lymphatics and the specific cellular origins of these cells. Hence, this study is potentially important for the fields of heart development and regeneration.

Comments for the author

While the manuscript contains huge amounts of data spanning an impressive list of mouse mutants and reporters, the conclusions are quite confusing and difficult to follow. At parts, it feels that the results are over-interpreted and that the data do not support the main conclusions of the paper. Overall, the authors try to answer two completely different questions: one regarding the role of macrophages in cardiac lymphangiogenesis, and the second one related to the origins of these

© 2021. Published by The Company of Biologists under the terms of the Creative Commons Attribution License (https://creativecommons.org/licenses/by/4.0/).

macrophages. I feel that in doing so, none of the questions is clearly addressed which significantly weakens the manuscript.
After carefully reading the manuscript, I am not sure however that adding experimental data will help improve this work, especially taking into account the global situation and the restricted ability to perform experiments. Thus, I'd suggest that the authors thoroughly revise the manuscript focusing mainly on data analysis and interpretation, tone down the main statements so that they reflect the actual data, shorten the introduction so that it can be clearly followed and in general, make this paper easy to follow. In addition, it will be essential that the authors provide control experiments as listed below and proper statistical analyses and quantification of phenotypes

Major problems:
1. The use of different macrophage markers (e.g. Csfr1+, Cxcr3cr1, Lyve1+, F4/80, CD68), clearly demonstrates the presence of phenotypically different macrophage populations in the heart (for example CXCR3+/Lyve1+ vs. Cxcr3+/Lyve1- as seen in Figure 1B-M, CD68+ seen in Figure 1Q and Lyve1+ in Figure 1U). The presence of distinct macrophage populations is even more evident in the depletion experiments (Figures 5 and 6). This fact however, is not elaborated at all in the interpretation of the results, that refers to all populations as a whole. Are these all populations overlapping? The authors should clarify this and should better characterize the distribution and quantify the expansion of the different populations at different developmental stages. Overall, if these are indeed different macrophage types (regardless of their origins), it is not clear at all which of these populations is important for cardiac lymphangiogenesis and at which stage?

2. The authors claim that cardiac resident macrophages derive from the yolk sac. However, clear quantification and statistics of the Tomato+ population are not provided. In this case also there are shortcomings in the interpretation of the data. The authors claim a modest but reproducible contribution of Csf1r-CreER+ cells to the lymphatic endothelium (based on Figure 2B, which shows 1 single Tomato+/Prox1+ cell) and conclude that there is no direct contribution of the Cxcr31+ of Flt3+ lineages. This could probably be explained by the broader and earlier expression of the Csfr1+ population? In any case the claim that a "subset of CSF1R+ EMP emerge from the yolk sac at E8.5 to colonize the developing heart and acquire an LEC phenotype" seems like a huge statement to be made from one double positive cell. In fact, one of the main strengths of this manuscript in my view is that it sort fo confirms that the macrophage/myeloid lineage does not contribute significantly to cardiac LECs...

3. The authors hypothesize that the effects of macrophages on LECs involve direct cell to cell interaction, eventhough they do not provide strong evidence supporting this claim. Throughout the text they claim several times that the macrophages appear to be "in close proximity or in direct contact" to lymphatic endothelial cells. However, images provided lack the resolution required to support this statement.

4. The patterning defects seen in the loss of function studies are difficult to interpret. The authors perform some experiments using DTA to deplete specific populations of macrophages but the images clearly show the presence of several macrophages (F5 E-H and F6 E-H). The authors should address this point. Was the treatment not efficient enough? Or was the treatment efficient but these macrophages belong to a different population and if so, what is their role? In addition, these experiments are performed at later time points raising questions about the specific timepoints at which macrophages are essential and for which function. Finally, the authors describe the malformations of the lymphatic vessels that many times catch up during development. Was there any dysfunction on the lymphatic vasculature or presence of edema at all.

5. The authors make use of an in vitro system based solely on the idea of a direct cell-cell interaction (see previous point about why this is problematic), to identify the requirement for macrophage HA for lymphatic sprouting. This result is not further confirmed in vivo and thus extrapolating from cultured CD68+ macrophages and iPSCs to the developing mouse heart, is problematic.

© 2021. Published by The Company of Biologists under the terms of the Creative Commons Attribution License (https://creativecommons.org/licenses/by/4.0/).

First revision

Author response to reviewers' comments

We thank the reviewers and editorial board for their informed comments and are pleased to have an opportunity to revise with new experiments to improve the findings of our study.

To strengthen our findings, we have now performed quantitative analyses of our genetic lineage tracing and cell ablation data arising from experiments using the *Cx3cr1*^{CreER} mouse, as well as providing further characterisation of the *Csf1r-Cre/ESR* and *Flt3*^{CreERT2} mice, as suggested by the reviewers. The new data is included in Figure 5 and Supplementary Figure 2. With regards to the use of the *Csf1r-Cre/ESR* mouse in particular and in line with the critique received we decided to remove the ablation data (previous Figure 5) and focused our analysis on a requirement for yolk sac-derived macrophages using the more (macrophage-) specific *Cx3cr1*^{CreER} line. We now include new data derived from ablation experiments using the *Cx3cr1*^{CreER} with tamoxifen pulsing at E8.5 (revised Figure 5 and 6), and lineage tracing downstream of tamoxifen pulsing at E12.5 (new Supplementary Figure 2), as requested. Lastly, we have revised the manuscript throughout to clarify and tone down specific sentences, as suggested by reviewers. These changes are highlighted in the point-by-point response below, with page and line numbers indicated.

A detailed response to the individual reviewer issues is provided below:

Reviewer 1 (Remarks to the Author):
In this manuscript, the authors characterize the role of tissue resident macrophages in the developing of the lymphatic system in the hearth using lineage tracing and loss of function experiments in vivo. This is a novel function for resident embryonic macrophages of yolk sac origin. The insights into the remodelling role of tissue resident macrophages in the embryo could be exploited to study this phenomenon in the adult during cardiovascular repair and disease. The work is very interesting even if I have some concerns regarding some tamoxifen inducing protocols and subsequent interpretation of the results.
We would like to thank the reviewer for this positive overall assessment of our study and suggestions for improvement.

1) This comment concerns the lineage tracing experiments in Figure 2, which is critical for the interpretation of further experiments. I am not convinced that the Csfr1-CreER and Cxrcr1-CreER alleles label completely non-overlapping sets of cells. In fact, Csfr1 EMP labelling obtained with 4OHT pulse at E8.5 can result in labelling of pre-macrophages as Csfr1+ EMPs acquire Cx3rcr1 expression later in development (Stremmel et al., 2018). This is especially relevant in the induction approach used here, as Tamoxifen persists longer in circulation than 4OHT. This would also explain the very similar phenotypes obtained with cell ablation of the two subsets. It would be helpful to have a more comprehensive characterization of the labelled cell subsets in E16.5 hearts and at shorter time points after Tamoxifen pulse induction as well, ideally with a method enabling quantification and analysis of multiple markers (e.g. flow cytometry).
We would like to clarify that both reporter lines, i.e. *Csf1r-CreER* and *Cx3cr1*^{CreER}, were used here to study the contribution of tissue-resident macrophages colonising the developing heart by the yolk sac. Thus, we agree with the reviewer that there is labelling of an overlapping population of yolk sac derived pre-macrophages with these reporters. However, the *Csf1r-Cre/ESR* reporter has a broader range via labelling of erythromyeloid progenitor cells, as the precursors of pre-macrophages and blood cells, as well as some endothelial cells in blood vascular and lymphatic beds, including the heart (Klotz et al. Nature 2015). To demonstrate this point we have now included data in new Supplementary Figure 2: in hearts collected at E12.5 from samples receiving a tamoxifen pulse at E8.5 (*Csf1r-CreER;tdTomato*), tdTomato+ cells are scattered throughout the subepicardial space and can either be LYVE1+ (macrophages) or LYVE1- (EMP derivatives). The new data and above clarification regarding the selection of the reporter lines are discussed in page 11, lines 230-237 of the revised manuscript. Since at the moment it is not possible to distinguish between *Csf1r* lineage derivatives fated to become macrophages versus (cardiac) lymphatic endothelial cells at a molecular level, we decided to remove the ablation data using this reporter line (previous Figure 5). Instead, we focused our analysis of the requirement for tissue-resident macrophages on cardiac lymphatics expansion exclusively using the macrophage-specific *Cx3cr1*^{CreER} reporter line and provide new data describing its efficiency in labelling (tdTomato) and deleting (DTA experiments) cardiac resident

© 2021. Published by The Company of Biologists under the terms of the Creative Commons Attribution License (https://creativecommons.org/licenses/by/4.0/).

macrophages, as well as comparing the ablation resulting from tamoxifen pulse at E8.5 versus E12.5. The new data are included in revised Figures 5 and 6, and Supplementary Figure 2, and are discussed in pages 12 (lines 244-246), 14 and 15 (lines 313-334) of the revised manuscript.

2) Related to the previous point. As in the genetic cell ablation studies in Figure 5 and 6 the Tamoxifen pulse is administered at a different time point to enable macrophage seeding of the heart (E12.5), it would be helpful to have an analogous qualitative and quantitative characterization of the cell subsets labelled with this induction strategy.

We have now included data detailing the efficiency in labelling and deletion achieved by the *Cx3cr1*^{CreER} line following tamoxifen pulses at E8.5 or E12.5. This has been included in revised Figure 5 and Supplementary Figure 2, and discussed in pages 12 (lines 244-246), 14 and 15 (lines 313-334) of the revised manuscript.

3) As the authors show that Flt3-derived cells are present in the E16.5 heart, does the genetic cell ablation of Flt3+ cells result in any detectable phenotype?

The *Flt3*^{CreERT2} line was used here to fate map definitive HSC blood cell contribution to tissue-resident macrophages in the developing heart (Figure 2). Ablation experiments using this line were not pursued because whilst we show that derivatives from this lineage are present in the heart at E16.5, their migration from the foetal liver to the developing heart takes place at a timepoint when cardiac lymphatic expansion is already well-established. In addition, the *Flt3*$^{+}$ lineage derivatives (tdTomato+) present in the heart at E16.5 lack the expression of LYVE1 and as such, do not appear to be tissue-resident macrophages (or at least LYVE1-expressing macrophages; new data in Supplementary Figure 2). These observations are supported by previous knock-out studies of Flt3 or Flt3 ligand which resulted in a mild phenotype, primarily affecting early lymphoid progenitors and did not delete macrophages or myeloid progenitors (e.g. Sitnicka E et al. Immunity 2002). The new data is discussed in page 12 (lines 261-264) of the revised manuscript.

4) What is the cellular mechanism by which lymphatic endothelial cells are lost in Pu.1 null and in macrophage-ablated hearts? It is shown that there is no difference in proliferation at E16.5. Have the authors checked an earlier time point (e.g. E14.5)? Are there signs of apoptosis?

The phenotype arising from macrophage depletion results from defects in LEC migration and patterning as supported by our quantitative assessment of the lymphatic network in the developing heart at E16.5, using the AngioTool software that revealed a significant reduction in total vessel length across the base-to-apex axis and laterally, and a decrease in vessel branching (Figures 3-6). LEC proliferation was initially considered as this has been suggested as the underlying cause of the dermal lymphatic phenotype reported in *Pu.1*$^{-/-}$ mice by Natasha Harvey's group (Gordon et al. Development 2010), but no gross changes were observed in proliferation levels in cardiac LECs. Our LEC-macrophage coculture studies suggest a role for macrophage HA in the regulation of LEC sprouting and future studies will aim to further dissect this molecular interaction *in vivo*.

Reviewer B (Remarks to the Author):

In this manuscript, Cahill and colleagues investigated lymphatic vessel patterning in the heart and studied the potential contribution of macrophages in this process. To do so they used 3 different genetic models to deplete macrophages and performed whole mount staining to evaluate and characterize the growing vasculature. Overall, the manuscript is well written and the scope is unambiguous, even if the contribution of macrophages in lymphangiogenesis has been widely studied and the data on hyaluronan-dependent action, although interesting are still too preliminary. In order to strengthen the conclusion and reach enough significance to the Development readership, I believe that several new key and control experiments would be required, especially the ones recommended below to refine the lineage tracing and depletion timing, as well as the quantification of the experiments already shown.

We would like to thank the reviewer for the detailed assessment of our study and suggesting experiments to strengthen our findings.

Major concerns:

Figure 1 A. The authors show the total count of CX3CR1+F4/80+ macrophages, which increases in time, suggesting active recruitment and/or proliferation of macrophages. Is the percentage of macrophages relative to total live cells remaining constant over the 3 time points, or does their presence become more dense at a certain stage?

© 2021. Published by The Company of Biologists under the terms of the Creative Commons Attribution License (https://creativecommons.org/licenses/by/4.0/).

We have revised the data presented in Figure 1, and now show the ratio of cardiac macrophages/total live singlet cells across the three developmental stages, rather than total cell counts. The flow cytometry data in combination with the immunostaining data also shown in Figure 1 support our hypothesis that from E12.5 there is a considerable increase in the number of tissue-resident macrophages in the heart.

To evaluate whether the increase in macrophage numbers is due to recruitment or/and macrophage proliferation the authors should perform an additional KI67 staining.
We respectfully disagree on the requirement for the suggested experiment, as our lineage tracing approaches in combination with the genetic ablation of macrophages (DTA studies) support the hypothesis of macrophage recruitment during heart development. Moreover, the main premise of the study is to show that tissue-macrophages associate with and influence the expansion and patterning of the lymphatic network during heart development, and not necessarily to dissect out the contribution of recruitment versus proliferation of macrophages in the developing heart.

- Fig 1Q: 2 different symbols should be used for the 2 types of interactions. Actually, it is not clear in the high mag showed in Fig1Q whether there is a branch or a leading edge. It seems rather an enlarged vessel segments. The authors should provide a better example.
Figure 1Q shows a magnified view of a VEGFR3+ lymphatic branching segment with CD68-GFP+ macrophages closely associated. With regards to examples of macrophages interacting or being in close association with leading edges of cardiac lymphatic vessels, these can be observed in Figure 1R-T and new Supplementary Figure 2A-C. In our data analysis, we do not have any indications to suggest a difference between macrophages associating with leading edges versus branches, and therefore, have not distinguished between the two.

- In the lines 206-208, the authors state that "tissue-resident macrophages colonize the embryonic heart prior to the formation of the main vascular networks and adopt a spatial distribution in close proximity to, and in contact with, the forming lymphatics." A quantification of the spatial distribution of the macrophages and an analysis of their preferential positioning close to lymphatic vessels is missing to support this claim.
We are unclear on the relevance of quantifying the spatial distribution of macrophages in the subepicardial space during cardiac lymphatic expansion. These cells colonise the epicardium-myocardium interface at early stages of heart development as described here and by others (Stevens SM et al. Dev Bio 2016) and given the close architecture of the tissue, any structures growing in this compartment such as blood and lymphatics vessels will naturally be in close proximity to macrophages. What we show in this study, through combined reporter-labelling, cell ablation studies and loss-of-function models, is that the close physical association of macrophages with lymphatic endothelium, rather than being serendipitous, regulates vessel expansion and patterning.

-line 255: the authors should show the data from a single injection (ie 13.5 and 15.5 separately) to understand when HSC start to effectively contribute macrophages to the heart. If it's just from E15.5, then HSC-derived macrophages would be unlikely to contribute to lymphatic vessel patterning. I think the authors should provide this piece of information by performing a time course of the tamoxifen pulse with this line.
The tamoxifen pulsing regimen for the experiments using the *Flt3*^{CreERT2} line was defined according to previous studies that thoroughly characterised *Flt3* expression during definitive haematopoiesis as well as the behaviour/efficiency of *Cre* lines driven by this locus to label myeloid cells (e.g. Epelman S et al. Immunity 2014). It is important to mention here that definitive haematopoiesis in the foetal liver is fully established only by E14.5 and recombination rates driven by *Flt3* are generally low during embryonic development, increasing postnatally to reflect the activation of the bone marrow as the main definitive haematopoiesis site. Therefore, to maximise the CreERT2 activity a multiple tamoxifen dosing was performed around the developmental stages when *Flt3* is active in the developing liver, in accordance with protocols in-place at our collaborator's laboratory, Professor Sten Eirik Jacobsen (Karolinska Institute). The data obtained *via* this approach revealed *Flt3*$^{+}$ lineage tdTomato^{+} derivatives present in the subepicardial space at E16.5 in the vicinity of the growing lymphatic network (Figure 2). However, upon closer investigation and combined labelling with an antibody against LYVE1, these cells were mostly negative for the expression of LYVE1 (new Supplementary Figure 2). As such, we agree with the reviewer's comment that it is unlikely that HSCs from the foetal liver contribute to the population of tissue-resident macrophages during the time-

© 2021. Published by The Company of Biologists under the terms of the Creative Commons Attribution License (https://creativecommons.org/licenses/by/4.0/).

window of cardiac lymphatic development onset. This information has now been included in the revised manuscript in pages 12 (lines 255-264).

- The authors state that "no direct contribution to cardiac lymphatic endothelium was observed.", however in Fig. 2J two double positive cells are visible, which might be on different planes but this cannot be excluded in the way the data are currently presented. Please explain adequately or show orthogonal views.

The data shown in Figure 2J has been analysed using orthogonal views and we provide here the images regarding the two cells mentioned by the reviewer. These are in close association with PROX1+ LECs, but are not PROX1+/tdTomato+. Thus, our statement is valid. Given the minor contribution of the *Flt3*^{CreERT2} line to the overall conclusions of our study, we have not included the orthogonal views (see below) in the revised manuscript.

We have removed unpublished data provided for the referees in confidence.

-Fig. 2I-L: macrophages traced with this line appear to be less close to lymphatic vessels compared to the previous 2 approaches. Could the authors quantify this? Moreover, for all three approaches, the authors should quantify the percentage of labelled macrophages over the total macrophage population.

Given the minor contribution of the *Flt3*^{CreERT2} line and evidence of very sparse labelling which do not impact on the overall conclusions of our study we do not feel there is a requirement to quantify in this instance.

- In Fig. 3, to exclude a possible bias in the quantification given by LYVE-1+ macrophages in close proximity to lymphatic vessels which are present in WT but absent in KO mice, a staining for another lymphatic vessel marker such as NRP2 would be required, otherwise provide in the materials and methods the quantification strategy that didn't take into account LYVE1+ macrophages.

Throughout this study the cardiac lymphatic vasculature was labelled using (at least) dual combinations of antibodies against LECs such as LYVE1/VEGFR3 or LYVE1/PROX1. This strategy allowed the combination of a third antibody to label coronary vessels (e.g. EMCN), therefore, maximising the information extracted/sample, and more importantly exclusion of the possible bias referred by the reviewer. It is important to mention also that our quantitative analyses of vascular networks (lymphatic and blood vessels) were done by tracing vessels-only using ImageJ and then analysing the traced networks on AngioTool. We have now provided more detail on the Material & Methods section regarding our quantitative approaches, page 28.

- In Fig. 3, I am not convinced that the length of the lymphatic vessel is reduced. The authors should clarify what they actually measured. In the example pictures, the vessels appear to reach a similar extension towards the apex in both the control and the mutant. Did the authors instead quantify the length of each vessel segment and plotted the sum? Information on how quantifications were made should be added also to the methods. The authors should rather find a way to quantify the most obvious phenotype at this stage, i.e. the increased caliber/diameter of the vessels.

We have now included further detail in the Material and Methods section regarding our quantitative analyses of vascular networks (page 28). AngioTool was used for these studies as it enables automated assessment of the entire network and measures the total vessel length covering the subepicardial surface, as well as vessel branchpoints. It is important to note that lymphatic vessels expansion takes place along two axes in a concurrent manner, base-to-apex and laterally, thus, total vessel length is a more adequate parameter to consider here. Moreover, according to our coculture studies macrophages influence LEC sprouting, meaning that disruption of macrophage-LEC interaction is expected to affect vessel length and branching. Proliferation was explored in our studies, but no obvious defects were observed in the *Pu.1*-deficient hearts.

- Please choose another representative picture in Fig. 3 for panel L and P, since PU.1-/- heart seems to have more branch points and similar length to the wt control.

We have now revised Figure 3 to include better representative images, as suggested by the reviewer.

-line 283: in order to exclude developmental delay in the mutants, the authors should compare other morphological parameters, e.g. the heart or the embryo size, the toes/fingers or the

© 2021. Published by The Company of Biologists under the terms of the Creative Commons Attribution License (https://creativecommons.org/licenses/by/4.0/).

whisker follicle development (see e.g. https://embryology.med.unsw.edu.au/embryology/index.php/Mouse_Stages). Is there any difference?

To avoid confusion, we have now deleted the end of this sentence, which now reads: "*A similar phenotype was observed at E19.5, before the onset of embryo demise at perinatal stages (Fig. 3K-T).*" and have noted instead that no gross changes were observed between mutant and control embryos in terms of size: "*Embryo size and heart morphology in Pu.1-deficient specimens appeared grossly normal, with no evident defects in heart growth, cardiac septation or compaction of the myocardial layer (Fig. S3E,F), hence ruling out the possibility of a secondary cardiac phenotype contributing to the observed lymphatic defects.*"

-Fig. S2E,F: the mutant heart shown appears much bigger, is this consistent. If yes, please discuss. The authors should provide a quantification. See also comment above.

As for the response above, no gross changes were observed in heart morphology and size in *Pu.1*-deficient mice versus control littermates.

- In the lines 287-289, the authors state that coronary blood vessels growth and patterning were mildly affected, however, the reduction in vessel length and number of junctions (Fig. 4I and J) is similar to the one observed for lymphatic vessels.

We apologise for the confusing sentence. The phenotype in coronary vessel growth and patterning was deemed mild because it was present at E16.5, but recovered by E19.5 potentially due to subsequent remodelling/trimming of the blood vascular bed. This phenomenon has been reported previously when assessing blood vascular development in the postnatal retina of mice lacking macrophages (Fantin et al. Blood 2010). We have revised the sentence; please refer to page 13, line 290.

- Fig. 4P and R shows a denser network of blood vessels in PU.1-/- mice, please quantify vessels number or replace the pictures.

In line with the changes made to Figure 3, the corresponding panels have also been replaced in Figure 4, since the same heart has been stained with antibodies against LYVE1 and EMCN. Please note that quantification of the subepicardial coronary network is provided in Figure 4 and showed no significant change between mutant and control hearts.

- line 294: the authors should mention that a similar experiment was done with op mice and compare it to theirs (Leid et al., 2016). Moreover, this figure is outside the scope of the manuscript but I agree it is important as control. The author should move it to supplementary.

We discuss the Leid et al study against our findings in page 20, line 452. Given the close proximity of tissue-resident macrophages to both vascular structures expanding in the epicardium-myocardium interface, we feel it is important to show and discuss both datasets in the main text.

- In lines 295-296 the authors refer to a paper previously published by Gordon et al. describing how skin macrophages are important to define dermal lymphatic vessels' caliber. The authors should evaluate if they also observe a similar difference in the skin. This would corroborate their data.

This evaluation was carried out and is shown here for the reviewer's benefit in this response: E16.5 skin preparation stained for EMCN and LYVE1. Since Gordon et al used the same *Pu.1*-deficient model and we are not assessing dermal lymphatic development, we feel there is no need to include these data in the manuscript.

We have removed unpublished data provided for the referees in confidence.

- Fig. S2G-N: the authors should quantify this experiment before discussing differences compared to other reports. Please provide it.

As shown in the representative panels provided in this figure, there are no obvious difference in the representation of PROX1^{+}/PH3^{+} cells in control versus mutant heart. Our statement does not impact on the findings reported by Gordon et al on their investigation of dermal lymphatic development, rather it highlights the inherent differences (heterogeneity) across different vascular beds in the same mutant embryo (*Pu.1*$^{-/-}$).

- line 308: in this case the authors should show the lineage tracing with R26R-tomato also when pulsed at E12.5, especially as the number of macrophages in fig 5 does not seem much reduced. It

© 2021. Published by The Company of Biologists under the terms of the Creative Commons Attribution License (https://creativecommons.org/licenses/by/4.0/).

could well be that at this stage the authors are lineage tracing and therefore depleting a higher than expected number of Csf1r+ EMP-derived LECs.
The authors should instead also provide the pulse at 8.5 as it will have more chances to affect a larger number of macrophages and they have already shown with the lineage tracing that targeting of Csf1r+ EMP-derived LECs is minimal. This approach will still be different from the pu.1 experiment, which is constitutive and where also non myeloid cells (e.g. B cell progenitors) will be affected.
We agree with the reviewer' concerns regarding the use of the *Csf1r-Cre/ESR* line in the ablation experiments. This line has a broader range of labelling erythromyeloid progenitor cells, the precursors of pre-macrophages and blood cells, as well as some endothelial cells in blood vascular and lymphatic beds, including the heart (Klotz et al. Nature 2015). To demonstrate this point we have now included data in new Supplementary Figure 2, showing in hearts collected at E12.5 from samples receiving a tamoxifen pulse at E8.5 (*Csf1r-Cre/ESR;tdTomato*), tdTomato+ cells are scattered throughout the subepicardial space and can either be LYVE1+ (macrophages) or LYVE1- (EMP derivatives). Since at the moment it is not possible to distinguish between *Csf1r* lineage derivatives fated to become macrophages versus (cardiac) lymphatic endothelial cells at a molecular level, we decided to remove the ablation data using this reporter line (previous Figure 5). Instead, we focused our analysis of the requirement for (yolk sac-derived) tissue-resident macrophages on cardiac lymphatics expansion exclusively using the macrophage-specific *Cxc3cr1*^{CreER} reporter line. We provide new data describing its efficiency in labelling (tdTomato) and deleting (DTA experiments) cardiac resident macrophages, as well as comparing the ablation resulting from tamoxifen pulse at E8.5 versus E12.5. The new data are included in revised Figures 5 and 6, and Supplementary Figure 2.

- lines 309-311: it is fundamental that the authors provide a quantification of the number of macrophages. From the pictures included in the figure it appears that the number of macrophages is not significantly affected in the mutants. How can the lymphatic phenotype be explained then if there is no significant reduction in macrophages? is the number of PROX1 nuclei any different?
As per our response to the previous point, these data have now been removed in the revised manuscript. However, the suggestion by the reviewer to quantify the number of macrophages has been carried out in the experiments using the *Cx3cr1*$^{CreER/+}$*;R26-DTA* model.

- line 329: Also in this case it is fundamental that the authors prove with a lineage tracing with R26Rtomato pulsed at E12.5 that the only cells labelled are macrophages and quantify the percentage over the total macrophage population.
The experiments suggested have been done and the new data shown in new Supplementary Figure 2 and discussed in page 15, line 321.

- line 333: it is fundamental that the authors provide a quantification of the number of macrophages.
The quantification of the number of macrophages is now included in revised Figure 5.

- line 339: in order to suggest that, the authors should verify that HSC-derived monocytes/macrophages in the heart are Cx3cr1 positive or not, either by antibody staining/FACS or by crossing the Cx3cr1 gfp reporter to the Flt3-creERT2;R26Rtomato. Moreover, the paper seems to lack the corresponding experiment of depleting HSC-derived macrophages with Flt3-Cre;R26R-DTA. To get a clear conclusion on what subpopulation of macrophages is required for heart lymphangiogenesis (YS-derived or HSC-derived) the authors should provide these data.
The *Flt3*^{CreERT2} line was used here to fate map definitive HSC blood cell contribution to tissue-resident macrophages in the developing heart (Figure 2). Ablation experiments using this line were not pursued because whilst we show that derivatives from this lineage are present in the heart at E16.5, their migration from the foetal liver to the developing heart takes place after the onset of cardiac lymphatic development. In addition, the *Flt3*-lineage derivatives (tdTomato^{+}) present in the heart at E16.5 lack the expression of LYVE1 and, as such, do not appear to be tissue-resident macrophages or at least LYVE1-expressing macrophages (new data in Supplementary Figure 2). These observations are supported by knock-out studies of Flt3 or Flt3 ligand which resulted in a mild phenotype, primarily affecting early lymphoid progenitors and did not delete macrophages or myeloid progenitors marked by *Flt3* expression (e.g. Sitnicka E et al. Immunity 2002). The new data is discussed in page 12 (line 261) of the revised manuscript.

© 2021. Published by The Company of Biologists under the terms of the Creative Commons Attribution License (https://creativecommons.org/licenses/by/4.0/).

- Why the numbers between Figure 4 and Figure 5 are so different? Please explain adequately.
We apologise for this mistake. Upon revision of the graphs shown in Figure 4 specifically the quantification of the number of junctions, we realised that an error had been made with the y-axis scale. This is now corrected.

- Fig. 7 A-K: to demonstrate that the observation the authors made means something, the authors should compare the tube formation rate without and with the addition of the macrophages. In addition, they should also use an independent cell line, such as fibroblast, to make sure that whatever difference is observed is macrophage-specific.
We respectfully disagree from the reviewer's assessment regarding the data in Figure 7A-K. The coculture model using human LECs and iPS-derived macrophages provides a unique opportunity to closely investigate and visualise cell-cell interactions through time-lapse imaging. Moreover, this model also enables the functional assessment of this interaction via specific LEC sprouting assays, as show in Figure 7L-X. Of note, these experiments were repeated several times and validated by using an additional human primary LEC line (purchased from Lonza) and independent human iPS-derived macrophages lacking a reporter gene expression ("wild-type"), with similar results being observed. This information has been included in the Material and Methods section, pages 26 and 27.

- Fig7L-P: no sprouting is visible in the no macrophage condition suggesting that it might not be a reliable model to study lymphatic sprouting in general. is sprouting occurring only in the presence of macrophages? if this is the case, it might still be used but it should be specified.
In the beads assay, sprouting is minimal in the absence of macrophages, but it significantly increases when macrophages are present suggesting that these cells support/simulate LEC sprouting. This is quantified with p-values in the revised text, page 17.

- Is the viability of HAase treated macrophages impaired?
No, HAase-treated macrophages exhibited similar cell viability as non-treated controls. This is now clarified in the revised text, page 18.

- In vitro experiments are not focusing on heart LVs and/or macrophages. Since, as the authors rightfully stated, both lymphatic vessels and macrophages from different locations possess peculiar characteristics, and that the findings from Gordon et al. on skin lymphatic vessels did not recapitulate what they saw in the heart, to strengthen their data it would be better to isolate both cell types from the embryonic heart and perform the same experiments. The authors should at least discuss this.
We thank the reviewer for this suggestion. The experiments involving isolation and co-culture of primary LECS and macrophages from embryonic hearts are not technically feasible. The caveat of distinct behaviour attributed to the source of cells has now been included in the Discussion section, page 22 (line 495).

Minor:
- line 40: what coincides with lymphatic emergence? macrophage recruitment?
This sentence has now been revised and reads: "*Here, we show that the distribution and prevalence of resident macrophages in the subepicardial compartment of the developing heart coincides with the emergence of new lymphatics and macrophages interact closely with the nascent lymphatic capillaries*".

- line 97: not precise. the authors cite a review where this process is well explained. HSCs are not liver-derived but AGM-derived but then reside in the liver. Please rephrase. Eg "...including fetal liver-resident EMPs and HSCs"
The sentence has been amended and now reads: "*In the embryo, macrophages arise initially from the extra-embryonic, transient yolk sac and subsequently from alternative sources within the embryo proper, including fetal liver-resident erythro-myeloid progenitors (EMP) and hematopoietic stem cells (HSC)*".

- line 100: not correct. the initial pool of macrophages is mainly replenished by monocytes derived from liver-resident EMPs, see the Ginhoux&Guilliams 2016 review
The sentence has been revised and now reads: "*Yolk sac-derived macrophages seed most tissue-resident niches, which are maintained through adulthood by self-renewal (e.g. microglia in the*

© 2021. Published by The Company of Biologists under the terms of the Creative Commons Attribution License (https://creativecommons.org/licenses/by/4.0/).

brain) or gradually replenished by fetal liver EMP-derived monocytes, with both populations having distinct roles in tissue injury responses".

- Please state the number of litters and animals used for all the figures presented. And fields analysed per sample in the materials and methods.
This information has been included in the revised figure legends and Material and Methods section, as requested.

- Rat polyclonal anti-mouse CD31 (clone MEC13.3) BD Pharmingen. Was it the monoclonal antibody MEC13.3 or was it used a different polyclonal one?
We apologise for this mistake and have now updated the details of the monoclonal antibody MEC13.3 in the key resources table (Material and Methods).

- Only + SEM is shown in all figure legends and not ± SEM, please correct it. The SEM is in general really high, it would be better to see individual values.
The ±SEM range is indicated in-text when discussing the results shown in the graphs.

- fig S1C: can the authors show across section (even virtual) through the specimen shown in S1C to better show where the macrophages reside within the cardiac tissue?
We thank the reviewer for this suggestion, but it has not been possible at this stage to provide sections of the specimens shown in Supplementary Figure 1C, or generate new ones. However, the representative images provided throughout the manuscript resulted from confocal imaging of whole-heart samples capturing the epicardial/subepicardial compartment where the macrophages reside during the developmental stages analysed. In addition, our observations are in agreement with previous reports, e.g. Stevens SM et al. Dev Bio 2016.

- Fig1. even though the images are beautiful, the authors should change the colour combination by showing LYVE1 in red and EMCN in blue (which is the least informative marker in this analysis), cause this will help the reader understand how many of the LYVE1+ macrophages are also CX3CR1+ and viceversa. Or add panels showing just the LYVE1 and CX3CR1 merge without using the white for LYVE1 to appreciate the colocalization. As it is shown at the moment it is difficult to appreciate which ones are the double positive macrophages. Especially at E12.5
We thank the reviewer for this suggestion. We have now changed the colour combination for the images shown in Figure 1B-M.

- Fig 1J-M: the authors should add 3 different symbols to indicate examples in the referred panel of these 3 different types of association.
We respectfully disagree with the reviewer regarding this suggestion. We feel that adding different symbols to capture macrophage interaction with specific parts of the vascular network would make the figure overly congested and difficult to interpret and would not provide relevant information; particularly given that there are presently no means to distinguish between such cells at a molecular level.

- line 198: CD68 gfp is shown only combined to VEGFR3. either rephrase or show gfp with lyve1 and prox1.
In this sentence, we discuss the data shown in Figure 1N-U, which resulted from the analysis of *hCD68-GFP* (Figure 1N-Q) and wild-type (Figure 1 R-U) mice. To make this point clearer, we have revised the preceding sentence to: "*To investigate this further we initially validated the findings in Cx3cr1$^{GFP/+}$ mice, by investigating the developing cardiac lymphatics in transgenic hCD68-GFP mice, which report enhanced GFP in macrophages under the control of the human CD68 promoter and enhancer sequences (Iqbal et al., 2014), and wild-type mice combined with VEGFR3, LYVE-1 or PROX1 markers at E16.5 (Fig. 1N-U).*"

- line 203: the authors cannot say that those macrophages are promoting fusions as this is a static analysis. please rephrase.
We have toned down this sentence. It now reads: "*Moreover, macrophages were detected bridging adjacent PROX1^{+} lymphatic tips potentially to promote vessel fusion (Fig. 1U, white arrowheads)…*".

-line 215: Csf1r is not expressed by the hemogenic endothelium but by the EMPs that emerge from such endothelium and also reside in the liver alongside dHSCs. Please correct.

© 2021. Published by The Company of Biologists under the terms of the Creative Commons Attribution License (https://creativecommons.org/licenses/by/4.0/).

We have now corrected this sentence, which reads: "*To define the origins of macrophages colonizing the developing heart during lymphatic vessel development, we employed genetic lineage tracing using Csf1r, Cx3cr1 and Flt3- based mouse models, which in combination capture the main hematopoietic sources from early to mid-gestation, i.e. the yolk sac (Csf1r and Cx3cr1) and fetal liver (Flt3)*".

- line 219: the authors should not use this abbreviation as it is confusing. CreER is a completely different transgene which was the base for the generation of the mutated version CreERT2. Please stick to the original line name.
The specific nomenclature of the transgenic lines is indicated in Material and Methods, key resource table, as well as in-text when discussed for the first time.

- line 221: most likely promoter. Please check the original paper
The sentence has been amended to: "*We initially employed the Csf1r-Mer-iCre-Mer transgenic model (henceforth referred to as, Csf1r-CreER mice) (Qian et al., 2011) crossed with R26R-tdTomato reporter mice in which CRE recombinase activity downstream of the Csf1r promoter sequence was induced by tamoxifen pulsing at E8.5 (Fig. 2A-D).*"

- line 232: most likely promoter. Please check the original paper
This sentence is correct, as the $Cx3cr1^{CreER}$ mouse is a knock-in model.

- line 240: if this is a knock in allele modification and not a transgene, then the allele modification should be written in superscript without hyphen
This has now been corrected.

- line 251: again modify line labelling according to the knockin allele
This has now been corrected.

- In Fig. S2B a representative KO mouse FACS plot should be shown.
As shown in now Supplementary Figure 3D, no $CD11b^+/F4/80^+$ cells were detected by flow cytometry in *Pu.1*-deficient hearts. Hence why no flow cytometry plot is shown for the KO.

- line 343: the main difference with Pu.1 mutants is more likely to be the fact that the Cre approach is inducible and far way from targeting all the macrophages as in pu.1. The authors should mention this.
We have now included this possibility in our discussion in page 15, line 335.

- In Fig. S3, not all macrophages are RFP positive. Are they selected by FACS before coculture?
No selection by FACS was done before coculture; the iPS-derived macrophages tend to progressively lose the expression of RFP when in culture. This however has no impact on their viability.

- In line 409-412, the authors state that macrophages in the developing heart lack VEGF-C expression, however, with the type of analysis provided in Fig. S4 this claim is a bit too strong. In order to prove this, the authors should either perform a FACS sorting of macrophages and perform a qPCR or perform an RNA-FISH staining for VEGF-C. Moreover, is VEGFC expression in the nucleus of LECs in panel F something expected? Any reference? Please rephrase or provide such information.
We have toned down this sentence, which now reads: "*Importantly, yolk sac-derived macrophages residing in the HA-rich layer of the epicardium/subepicardium compartment of the developing mouse heart appear to lack VEGF-C expression (…)*". Regarding the expression pattern of VEGF-C, two different antibodies were tested, and both revealed immunoreactivity in the nucleus as well as diffused cytoplasmic and extracellular staining. Nuclear staining for members of the VEGF family, including VEGF-C has been reported previously, e.g. Shin Y-J et al. Journal Histochemistry and Cytochemistry 2014 or Zhang Q et al. Arthritis Research and Therapy 2007, but the functional implications of such localization remain poorly understood.

- The authors claim that macrophages directly affect lymphatic vessel growth in the heart during development, however, besides blood vessels, they did not check and/or exclude the possible contribution of other cell types, which could induce lymphangiogenesis, and might be affected by macrophage depletion. The authors should comment on this possibility in the discussion.

© 2021. Published by The Company of Biologists under the terms of the Creative Commons Attribution License (https://creativecommons.org/licenses/by/4.0/).

We are unclear as to which other cell types may induce lymphangiogenesis? Our study identifies the macrophage-LEC cell-cell interactions taking place in the subepicardial compartment during cardiac lymphatic expansion as being essential for proper growth and patterning of the network. Coronary blood vessels, which form in the same space, also associate closely to tissue-resident macrophages and rely on this cell-cell interaction to grow and pattern, at least at initial stages of heart development (Figure 4 and the previous study Leid et al., Circulation Research 2016). Finally, our *in vitro* co-culture and loss of HA-function experiments support a direct interaction between hiPSC-derived macrophages and hLECs mediated by HA in the absence of other cell types.

Reviewer 3 (Remarks to the Author):
The studies described in this paper characterize the role of tissue resident macrophages in the formation and remodeling of cardiac lymphatic vessels. The authors characterize malformations in cardiac lymphatics upon macrophage ablation in different mouse backgrounds during development. This work expands on previous publications, including from the same lab, analyzing the role of macrophages in lymphatic development. Here, the main focus is the contribution/role of organ-specific (heart)/tissue resident macrophages during the formation of cardiac lymphatics and the specific cellular origins of these cells. Hence, this study is potentially important for the fields of heart development and regeneration.
While the manuscript contains huge amounts of data spanning an impressive list of mouse mutants and reporters, the conclusions are quite confusing and difficult to follow. At parts, it feels that the results are over-interpreted and that the data do not support the main conclusions of the paper. Overall, the authors try to answer two completely different questions: one regarding the role of macrophages in cardiac lymphangiogenesis, and the second one related to the origins of these macrophages. I feel that in doing so, none of the questions is clearly addressed, which significantly weakens the manuscript.
After carefully reading the manuscript, I am not sure however that adding experimental data will help improve this work, especially taking into account the global situation and the restricted ability to perform experiments. Thus, I'd suggest that the authors thoroughly revise the manuscript focusing mainly on data analysis and interpretation, tone down the main statements so that they reflect the actual data, shorten the introduction so that it can be clearly followed and in general, make this paper easy to follow. In addition, it will be essential that the authors provide control experiments as listed below and proper statistical analyses and quantification of phenotypes.
Major problems:
1. The use of different macrophage markers (e.g. Csfr1+, Cxcr3cr1, Lyve1+, F4/80, CD68), clearly demonstrates the presence of phenotypically different macrophage populations in the heart (for example CXCR3+/Lyve1+ vs. Cxcr3+/Lyve1- as seen in Figure 1B-M, CD68+ seen in Figure 1Q and Lyve1+ in Figure 1U). The presence of distinct macrophage populations is even more evident in the depletion experiments (Figures 5 and 6). This fact however, is not elaborated at all in the interpretation of the results that refers to all populations as a whole. Are these all populations overlapping? The authors should clarify this and should better characterize the distribution and quantify the expansion of the different populations at different developmental stages. Overall, if these are indeed different macrophage types (regardless of their origins), it is not clear at all which of these populations is important for cardiac lymphangiogenesis and at which stage?
We thank the reviewer for the detailed assessment of our study, constructive criticism and suggested experiments to improve it.
As the reviewer states, our lab retains an interest in understanding the cellular and molecular events underpinning the expansion of cardiac lymphatics, as a developmental process and as might be therapeutically be explored to enable tissue repair in the context of myocardial infarction in the adult heart. To this end, we have previously investigated the origins of cardiac LECs and now focused on the cell-cell interactions taking place during lymphangiogenesis. This is important given that cardiac lymphatics, as well as coronary blood vessels, initially grow in the subepicardial space, which is densely colonised by macrophages from E10.5 (Supplementary Figure 1), and as such, as the vessels navigate the dense epicardium-myocardium interface along the base-to-apex axis and laterally to cover the entire myocardial surface, LECs will interact with these cells. The main question we are trying to address here is what is the relevance of such interaction (e.g. Figure 1)? Having demonstrated that macrophages are essential for cardiac lymphatics growth and patterning (Figures 3 and 5) the next step was to characterise their origin. The former is not a trivial task as despite using three different transgenic approaches it has proven difficult to come to a definitive conclusion as to the exact origin of <u>all</u> the tissue resident macrophages during heart development. Having said

© 2021. Published by The Company of Biologists under the terms of the Creative Commons Attribution License (https://creativecommons.org/licenses/by/4.0/).

that, at E12.5 when cardiac lymphatic development initiates, the main contribution to tissue-resident macrophages comes from the yolk sac as demonstrated by our lineage tracing and use of the reporter *Cx3cr1*^{GFP} (Figures 1 and 2, and new Supplementary Figure 2), therefore, we can implicate yolk sac-derived macrophages in this process. With regards to non-yolk sac-derived macrophages (i.e. fetal liver), these do not appear to be relevant for the onset of cardiac lymphatic expansion, but we cannot categorically rule out a role for these cells at later developmental stages (e.g. maintenance versus remodelling of the vascular network). This has been discussed in page 20 (line 446) of the revised manuscript.

To further dissect the cardiac macrophage phenotype at a molecular level, as suggested by the reviewer, we would have to apply unbiased scRNA-seq followed by careful investigation of marker analysis *in situ*, mapped against lymphatic expansion, and selective cell targeting, based on their marker profile. We feel that such approach that we now discuss in the revised Discussion section (see page 22, line 502), requires extensive and lengthy new experiments and analyses, which are beyond the scope of the current study. Also, it is worth mentioning that even when using the above approaches similar problems and concerns as the ones raised by the reviewer have been experienced by others tracking the origin of myeloid cell populations in adult murine tissues, e.g. atherosclerotic tissues (Lin J-D et al. JCI insight 2019).

We hope that extensive changes to the revised text including various clarifications on the use of the distinct lineage tracing models; a focus on tissue-resident macrophages via *Cxc3cr1*^{CreER} based cell ablation rather than *Csf1r*- targeting (see response below) and toning down of previous claims will focus the study and make interpretation of the major findings much clearer.

2. The authors claim that cardiac resident macrophages derive from the yolk sac. However, clear quantification and statistics of the Tomato+ population are not provided. In this case also there are shortcomings in the interpretation of the data. The authors claim a modest but reproducible contribution of Csf1r-CreER+ cells to the lymphatic endothelium (based on Figure 2B, which shows 1 single Tomato+/Prox1+ cell) and conclude that there is no direct contribution of the Cxcr31+ of Flt3+ lineages. This could probably be explained by the broader and earlier expression of the Csfr1+ population? In any case the claim that a "subset of CSF1R+ EMP emerge from the yolk sac at E8.5 to colonize the developing heart and acquire an LEC phenotype" seems like a huge statement to be made from one double positive cell. In fact, one of the main strengths of this manuscript in my view is that it sort fo confirms that the macrophage/myeloid lineage does not contribute significantly to cardiac LECs...

Both *Csf1r-Cre/ESR* and *Cx3cr1*^{CreER} reporter lines were used here to study the contribution of tissue-resident macrophages colonising the developing heart by the yolk sac. This approach led to labelling of an overlapping population of yolk sac derived pre-macrophages. However, the *Csf1r-Cre/ESR* reporter has a broader range in labelling erythromyeloid progenitor cells, the precursors of pre-macrophages and blood cells, as well as some endothelial cells in blood vascular and lymphatic beds, including the heart (Klotz et al. Nature 2015). To demonstrate this point we have now included data in new Supplementary Figure 2, showing in hearts collected at E12.5 from samples receiving a tamoxifen pulse at E8.5 (*Csf1r-Cre/ESR;tdTomato*), tdTomato+ cells are scattered throughout the subepicardial space and can either be LYVE1+ (EMP-derived macrophages) or LYVE1- (EMPs or non-macrophage EMP derivatives). The new data and above clarification regarding the selection, as well as the limitations of the reporter lines are discussed in page 11 (line 230-236) of the revised manuscript. Also, the statement indicated by the reviewer has been toned down to reflect the latter clarification; please refer to page 11 (line 223).

Since at the moment it is not possible to distinguish between *Csf1r* lineage derivatives fated to become macrophages versus (cardiac) lymphatic endothelial cells at a molecular level, we decided to remove the ablation data using this reporter line (previous Figure 5). Instead, we focused our analysis of the requirement for tissue-resident macrophages on cardiac lymphatics expansion exclusively using the macrophage-specific *Cxc3cr1*^{CreER} reporter line and provide new data describing its efficiency in labelling (tdTomato) and deleting (DTA experiments) cardiac resident macrophages, as well as comparing the ablation resulting from tamoxifen pulse at E8.5 versus E12.5. The new data are included in revised Figures 5 and 6, and Supplementary Figure 2.

3. The authors hypothesize that the effects of macrophages on LECs involve direct cell to cell interaction, even though they do not provide strong evidence supporting this claim. Throughout the text they claim several times that the macrophages appear to be "in close proximity or in

© 2021. Published by The Company of Biologists under the terms of the Creative Commons Attribution License (https://creativecommons.org/licenses/by/4.0/).

direct contact" to lymphatic endothelial cells. However, images provided lack the resolution required to support this statement.

We respectfully disagree with the reviewer on the assessment of our imaging data, which coupled with orthogonal views as shown in Figure 1 clearly support our conclusion that macrophages and LECs closely associate during cardiac lymphatic development. Moreover, the quantitative analyses of hearts deficient in tissue-resident macrophages further support this conclusion by revealing a significant reduction in vessel length (growth) and branching (patterning).

4. The patterning defects seen in the loss of function studies are difficult to interpret. The authors perform some experiments using DTA to deplete specific populations of macrophages but the images clearly show the presence of several macrophages (F5 E-H and F6 E-H). The authors should address this point. Was the treatment not efficient enough? Or was the treatment efficient but these macrophages belong to a different population and if so, what is their role? In addition, these experiments are performed at later time points raising questions about the specific timepoints at which macrophages are essential and for which function. Finally, the authors describe the malformations of the lymphatic vessels that many times catch up during development. Was there any dysfunction on the lymphatic vasculature or presence of edema at all.

We have now included data detailing the efficiency in deleting macrophages achieved by the *Cx3cr1*^{CreER} line following tamoxifen pulses at E8.5 or E12.5. This has been included in revised Figure 5 and Supplementary Figure 2, and discussed in page 15. The E16.5 timepoint was selected here, because at this developmental stage the cardiac lymphatic should already be growing towards the apex of the developing heart, as well as branching laterally to cover a wide area of the ventricular surface. The latter enables robust and reproducible quantification of different vascular parameters. It is also worth mentioning that cardiac lymphatics only start developing from E12.5 onwards, with inherent developmental delays across samples being a confounding factor for assessment of the vasculature at intermediate timepoints.

With regards to a general phenotype of the mutants analysed here, we have not observed any gross differences between control and macrophage-deficient embryos in terms of size or appearance (e.g. haemorrhaging). Oedema and bleeding are normally associated to defects impairing cardiac development/function and vessel wall integrity. In our model, we have not observed evidences for cardiac defects or a role for tissue-macrophages stabilising the vascular wall (e.g. LEC-LEC junctions). Instead, our data suggest a role for macrophages in growth and branching.

5. The authors make use of an in vitro system based solely on the idea of a direct cell-cell interaction (see previous point about why this is problematic), to identify the requirement for macrophage HA for lymphatic sprouting. This result is not further confirmed in vivo and thus extrapolating from cultured CD68+ macrophages and iPSCs to the developing mouse heart, is problematic.

As discussed above, our data clearly supports a role for macrophage-LEC interactions and the use of a coculture system here enabled the identification of HA as a potential molecular player. We agree that these findings need to be explored further by modulating macrophage HA *in vivo*. However, such approaches will require tools not currently available (e.g. mice carrying the "floxed" HA synthase isoform alleles). We have revised the discussion to include the need for future experiments to validate the role of macrophage HA in cardiac lymphatic development (please refer to page 22). Lastly, we also recognise an indirect effect, *via* the release of a soluble growth factor(s) by tissue-resident macrophages, cannot be categorically ruled out at this stage, albeit we can discount VEGF-C given the lack of expression by macrophages in the developing heart. This acknowledgement has been included in the Discussion section, page 22.

<u>Second decision letter</u>

MS ID#: DEVELOP/2020/194563

MS TITLE: Tissue-resident macrophages regulate lymphatic vessel growth and patterning in the developing heart

© 2021. Published by The Company of Biologists under the terms of the Creative Commons Attribution License (https://creativecommons.org/licenses/by/4.0/).

AUTHORS: Thomas J Cahill, Xin Sun, Christophe Ravaud, Cristina Villa del Campo, Konstantinos Klaourakis, Irina-Elena Lupu, Allegra M Lord, Cathy Browne, Sten Eirik W. Jacobsen, David R Greaves, David G Jackson, Sally A Cowley, William James, Robin P Choudhury, Joaquim Miguel Vieira, and Paul R. Riley
ARTICLE TYPE: Research Article

I am happy to tell you that your manuscript has been accepted for publication in Development, pending our standard ethics checks. One reviewer had additional comments regarding some of the figures, but having looked at them carefully, my evaluation is that additional work would be counter-productive and wold not alter the conclusions of the work.

Reviewer 1

Advance summary and potential significance to field

The authors have addressed all the points and questions raised in the previous review cycle.

Comments for the author

No additional comment

Reviewer 2

Advance summary and potential significance to field

The authors have made some effort to further advance their work. While this has undoubtedly improved the manuscript, some of the raised issues were still not satisfactorily addressed.

Comments for the author

- Reviewer's original comment: Fig. 2I-L: macrophages traced with this line appear to be less close to lymphatic vessels compared to the previous 2 approaches. Could the authors quantify this? Moreover, for all three approaches the authors should quantify the percentage of labeled macrophages over the total macrophage population.

Authors' response: Given the minor contribution of the Flt3CreERT2 line and evidence of very sparse labeling which do not impact on the overall conclusions of our study we do not feel there is a requirement to quantify in this instance.

Reviewer's new comment: the authors only addressed the comment about the Flt3CreERT2 line, however, they completely neglected the second part of the comment: "Moreover, for all three approaches, the authors should quantify the percentage of labeled macrophages over the total macrophage population." This is particularly important especially for the Cx3cr1-cre line that is later used for macrophage depletion.

- Reviewer's original comment: Please choose another representative picture in Fig. 3 for panel L and P, since PU.1-/- heart seems to have more branch points and similar length to the wt control.

Authors? response: We have now revised Figure 3 to include better representative images, as suggested by the reviewer.

Reviewer's new comment: Also in this case the PU.1-/- heart does not seem to have less lymphatic branch points, especially on the dorsal side at both low and high mag (Fig. 3K, L, O and P). While the ventral high mag picture do show less junctions in the mutant (Fig. 3N and R), this does not seem obvious at all at the low mag where the whole plexus is included in the view (Fig. 3M and Q). Hence, it is not clear whether the quantifications are done on the ventral or dorsal side and the size of the area quantified. Even considering the whole heart surface the lymphatic plexus of the hearts shown in Fig. 3K,L,M and N seems to be far away from the 400 junctions quantified in Fig. 3T

© 2021. Published by The Company of Biologists under the terms of the Creative Commons Attribution License (https://creativecommons.org/licenses/by/4.0/).

with angiotool. Please provide a representative snapshot of the Angiotool quantification of one of the panels shown in fig3 showing what has actually been counted as juntion. At least as a reviewer figure.
Moreover the authors mentioned in their response that "Throughout this study the cardiac lymphatic vasculature was labeled using (at least) dual combinations of antibodies against LECs such as LYVE1/VEGFR3 or LYVE1/PROX1." If this is the case, the authors have to show a dual lymphatic staining for figure 3 (and 5), as otherwise with the current labeling strategy that labels both lymphatic ECs and macrophages it is almost impossible to discern a small lymphatic sprout from a macrophage interacting with lymphatic vessels. See for example Fig.3L,N.
Alternatively, or in addition, the authors could superimpose an outline of the lymphatic plexus to the immunofluorescence picture, for example using a dashed white line.

- Reviewer's original comment: Fig. S2G-N: the authors should quantify this experiment before discussing differences compared to other reports. Please provide it.

Authors? response: As shown in the representative panels provided in this figure there are no obvious difference in the representation of PROX1+/PH3+ cells in control versus mutant heart. Our statement does not impact on the findings reported by Gordon et al on their investigation of dermal lymphatic development rather it highlights the inherent differences (heterogeneity) across different vascular beds in the same mutant embryo (Pu.1-/-).

Reviewer's new comment: PU.1-/- embryos are supposed to have an impaired lymphatic vessel growth in terms of vessel length and number of junctions; the fact that the authors include the same number of arrows to indicate pH3 positive LECs suggests a different proliferation in wt and macrophage depleted mice.
Proliferation of LECs was not quantified and should be provided as percentage of pH3 positive PROX1 nuclei. Moreover, in the new supplementary figure 3 G-N pictures lymphatic vessels seem comparable in terms of length and branching between the 2 different genotypes. Even if the authors statement doesn't impact previous findings reported by Gordon et al because differences exist in different vascular beds, the authors cannot conclude that there is no difference in proliferation if just a picture of 1 wt versus one mutant is shown and no quantification is provided. Quantification is hence required.

© 2021. Published by The Company of Biologists under the terms of the Creative Commons Attribution License (https://creativecommons.org/licenses/by/4.0/).
